# A potent estrogen receptor and microtubule specific purine-benzothiazole-based fluorescent molecular probe induces apoptotic death of breast cancer cells

**DOI:** 10.1038/s41598-022-12933-8

**Published:** 2022-06-24

**Authors:** Surajit Barman, Subhajit Ghosh, Rajsekhar Roy, Varsha Gupta, Satyajit Ghosh, Surajit Ghosh

**Affiliations:** 1 Organic and Medicinal Chemistry and Structural Biology and Bioinformatics Division, CSIR-Indian Institute of Chemical Biology, West Bengal, Kolkata, 700 032 India; 2 Department of Bioscience & Bioengineering, Indian Institute of Technology Jodhpur, Karwar, Rajasthan 342037 India; 3 Academy of Scientific and Innovative Research (AcSIR), Ghaziabad, 201002 India

**Keywords:** Chemistry, Medicinal chemistry, Drug discovery

## Abstract

Breast cancer is the most common malignancy in women and is a heterogeneous disease at molecular level. Early detection and specificity are the key prerequisite for the treatment of this deadly cancer. To address these issues attention on the breast cancer specific receptor protein(s) is the most realistic option. Herein estrogen (E) and progesterone (Pg) receptors(R) were considered to design fluorescent molecular probes with possible therapeutic option. We adopted QSAR technique to design a library of benzothiazole-purine hybrid molecules. Molecular docking offers us three screened molecules as most potential. Among these molecules one abbreviated as “CPIB” showed blue fluorescence and detected ER positive cancer cells at 1 nM concentration. At elevated concentration, CPIB induces apoptotic deaths of same cancer cells through targeting intracellular microtubules without affecting normal cells or ER negative cells. CPIB is one of its kind with two-in-one potential of “Detection and Destroy” ability targeting ER positive breast cancer cells.

## Introduction

The early detection of breast cancer is essential for the effective therapy and improvement of the patient. Although being useful, orthodox detection procedures such as CT scan, MRI, radioisotope imaging and angiography uses components that are always emission active, thus always keeps producing signal and have been termed as “always on”^1^. Even the compounds lack behind in the affinity and selectivity towards the target. To overcome these shortcomings in recent years scientists have developed fluorescent probes that could be “turned on” in specific circumstances or only upon some specific interactions^2^. These designs can be modulated in such a way that therapeutic efficacy could be included against the malignant cells. In this regard, to design and develop a small molecule that could have both the characteristics, i.e., detectability, and anti-proliferative activity, bottom up approach is the best possible way one can follow. While designing therapeutically active fluorescence probes against breast cancer, the first hurdle that comes up is the ability to detect the malignant cells. There are numerous molecular imaging probes reported for breast cancer diagnosis and therapy^3^, but only a few of them (mainly PET tracers) have entered the clinical trials^4,5^. The major risk factors for breast cancer are linked to hormone exposure^6^. Estrogen is clearly a promoter of breast cancer, through its binding of the estrogen receptor (ER) situated in the nucleus [determined by Estrogen Receptor 1(ESR1)], a ligand-triggered transcription factor^7^. During the menstrual cycles, an imbalance between estrogen and progesterone boosts cell proliferation and may cause DNA damage. The recurrence of the process at every cycle, an error prone repair process may occur, leading to mutations in pre-malignant, and later on in malignant cells^8^. On this stage, estrogen enhances the growth of these cells and the proliferation of stromal cells that promotes cancer development^9^. Target of these receptor is considered as one of the most vital molecular targets for the establishment of specialized breast cancer drugs and markers to the researchers. On the other hand, microtubule is considered as one of the key intracellular target for anticancer therapeutic development. Slight perturbation in microtubule dynamicity, affects the important intracellular trafficking, slower down the cell signalling, inhibits cell cycle and induces cellular invasion and migration^10^. Still, many of the microtubule targeting molecules shows limitations, among these resistance and tumor recurrence are the deadly examples^11^. Although, microtubule targeting molecules got success in clinical applications, use as regime for complete anticancer therapy still to be addressed^12^. Thus, development of a suitable microtubule targeting molecule with proper characterization is always warranted. Any tubulin targeted small molecules, which inhibit the microtubule dynamicity, have huge potential to act as an anti-cancer agent having less cytotoxicity against normal cells^13^.

Heterocyclic scaffolds like benzothiazole, purine, quinoline and pyrimidine are served as wide range of biological activity. A Hybrid system of these heterocyclic moiety is basically designed to interact with multiple targets or to amplify its effect through action on another bio-target as one single molecule and as agents modulating more than one target could have superior efficacy compared to single target drugs^14^. We have concomitantly designed this benzothiazole-based hybrid molecule to address more than one biological target for cancer treatment^15^. Thus, this strategy will offer dual effect like identification and killing of cancer cell. Although, many benzothiazole derivatives have been reported as anti-cancer agents but their activity is limited to in vitro level only^16^. As it is very important to consider the side effects of the molecules for the clinical trial^17^. To overcome the issue of non-specificity for imaging and anticancer activity, estrogen receptor and microtubule targeted fluorescent probe design is effective way to reach the goal^18^. Three breast cancer cell lines were chosen to represent the three most well defined sub-types of breast cancer. MDA-MB-231 is a basal-like cell line representing triple-negative breast cancer. SkBr3 is a HER2-like cell line representing HER2 overexpressing breast cancer. MCF-7 is a luminal-like cell line representing estrogen receptor positive breast cancer^19^. An additional cell line, MCF-10A normal epithelial mammary gland of breast was used as a control.

In the current study, using 2D-QSAR approach we have designed library of 15 molecules containing benzothiazole analogues that can detect breast cancer cell and inhibit its proliferation. 2D-QSAR method was used to postulate the target ability of the molecules towards breast cancer cells, which showed to be more specific towards ER compared to PgR of the cell surface. Furthermore, studies showed 6 best fitted of these 15 molecules were taken further to perform molecular docking, which showed CPIB, Q-CPIB and M-CPIB to be the prime molecules that targets ER specifically. From the designed molecules we found at 1 nM concentration, CPIB could specifically and selectively detect MCF-7 cells. A strong fluorescence signal of CPIB was observed at mentioned concentration in the MCF-7 cell lines, whereas no signal in other mentioned cancer and normal cell line under the microscope. The specific imaging properties were checked in vitro as well as in 3D spheroid culture. The results clearly demonstrate that CPIB can be used as a fluorescent probe to selectively detect ER positive breast cancer (MCF-7) cells. We found that CPIB binding close to the colchicine pocket of the tubulin and also perturbing the microtubule dynamics supported by various experiment like microtubule assembly assay (DAPI) and FRET experiment. Next, we have performed various cellular studies to understand the detail mechanism of biological activity. Our study also demonstrates that our newly designed molecular probes affect microtubule dynamics and confers extensive cytotoxicity, which eventually leads to arrest the cell cycle progression and cell death in MCF-7 cancer cells. These dual properties of the molecule have been mechanistically analysed in different cancer cell lines. Finally, the tumor inhibitory effect has been analysed in more aggressive 3D tumor model of cancer cells.

## Results and discussion

### Creation of A benzothiazole-based 2D-QSAR model for breast cancer targeted therapeutics

The most prominent hormone receptors that are present on the surface of breast cancer cells are estrogen receptor (ER) and progesterone receptor (PgR)^20^. Thus, targeting these hormone receptors for therapeutic purposes is indeed a relevant approach to design therapeutics against breast cancer. In the recent understanding, benzothiazole moiety is considered as one of the key pharmacophore unit in various therapeutic chemical hits^21^. Considering this important role of benzothiazole core, here we have designed several benzothiazole-based molecules and constructed a 2D-QSAR model targeted ER and PgR for screening those molecules. Basically the screening is done on the basis of affinity and efficacy of the molecule towards ER and PgR.

To meet our goal, 30 ER targeting molecules were selected from literature and they were divided into training set and test set keeping 80% of the molecules in the training set^22^. The same modeling was performed in case of previously reported 30 PgR specific molecules^23^. Through partial least square (PLS) model keeping IC50 of the molecules as dependent property we generated a relevant correlation coefficient corresponding to both the receptors. Then the 2D QSAR model validation was further validated through leave one out method. The correlation coefficient (R^2^) was calculated to be 0.989 and 0.981 respectively for ER and PgR (Supplementary Fig. 1) thus validating the model. Next, a small library containing fifteen molecules was constructed and were subjected to validate though this QSAR model (Supplementary Fig. 2). For the new compounds nine principal descriptor components (AlogP, Molecular_Weight, Number of H-donors, Number of H-acceptors, Number of rotatable bonds, Number of aromatic rings, and Molecular_Fractional polar surface-area) were correlated and were used to generate Partial least-square (PLS) approaches contemplating 2D-QSAR validation. A statistical linear correlation of the nine descriptors were taken as dependent variable to generate the PLS score.

The correlation model depending on the nine descriptors:

$$ {\text{Correlation}}\;{\text{ Model}} = - {7}.{6}0{96} - {5}.{\text{6655e}} - 00{2}*\left[ {{\text{AlogP}}} \right] + 0.000{28867}*\left[ {{\text{Molecular}}\_{\text{Weight}}} \right] + \, 0.0{416}0{4}*\left[ {{\text{Num}}\_{\text{H}}\_{\text{Donors}}} \right] + 0.0{89}0{57}*\left[ {{\text{Num}}\_{\text{H}}\_{\text{Acceptors}}} \right] + \, 0.0{54865}*\left[ {{\text{Num}}\_{\text{RotatableBonds}}} \right] - \, 0.{37762}*\left[ {{\text{Num}}\_{\text{Rings}}} \right] + { 3}.{6}0{88}*\left[ {{\text{Num}}\_{\text{Aromatic}}\;{\text{Rings}}} \right] + { 2}.0{4}*\left[ {{\text{Molecular}}\_{\text{Fractional}}\;{\text{polar}}\;{\text{surface}}\;{\text{area}}} \right] $$

Model with the square correlation coefficient (R^2^) value of ≥ 0.9 were considered as measurement of good fit, as the cross-validated squared correlation coefficient (q^2^) that was predicted using “leave one out” (LOO) method, should be high as a good indicator for predicting the power of the QSAR model, and the difference between this and R^2^ should not be more than 0.3. The generated model showed, for the final six molecules, a good value of square correlation coefficient (R^2^) i.e., 0.995 for ER receptors (Supplementary Fig. 1) and value of cross-validated squared correlation coefficient (q^2^) to be 0.805 defining the good efficacy against ER receptors of the designed molecules. In case of PgR the value of square correlation coefficient (R^2^) came out to be 0.464, showing a comparatively lower efficacy of the compounds against PgR. The prevalent mode of interactions was also checked and summarized in favour of ER compared to PgR (Supplementary Fig. 1). The outcome of this model shows that molecules are more prone towards ER than PgR, further molecular docking validation could be performed to further screen the molecules for receptor specificity between ER and PgR.

### Screening of molecules using molecular docking studies

**Figure 1:**
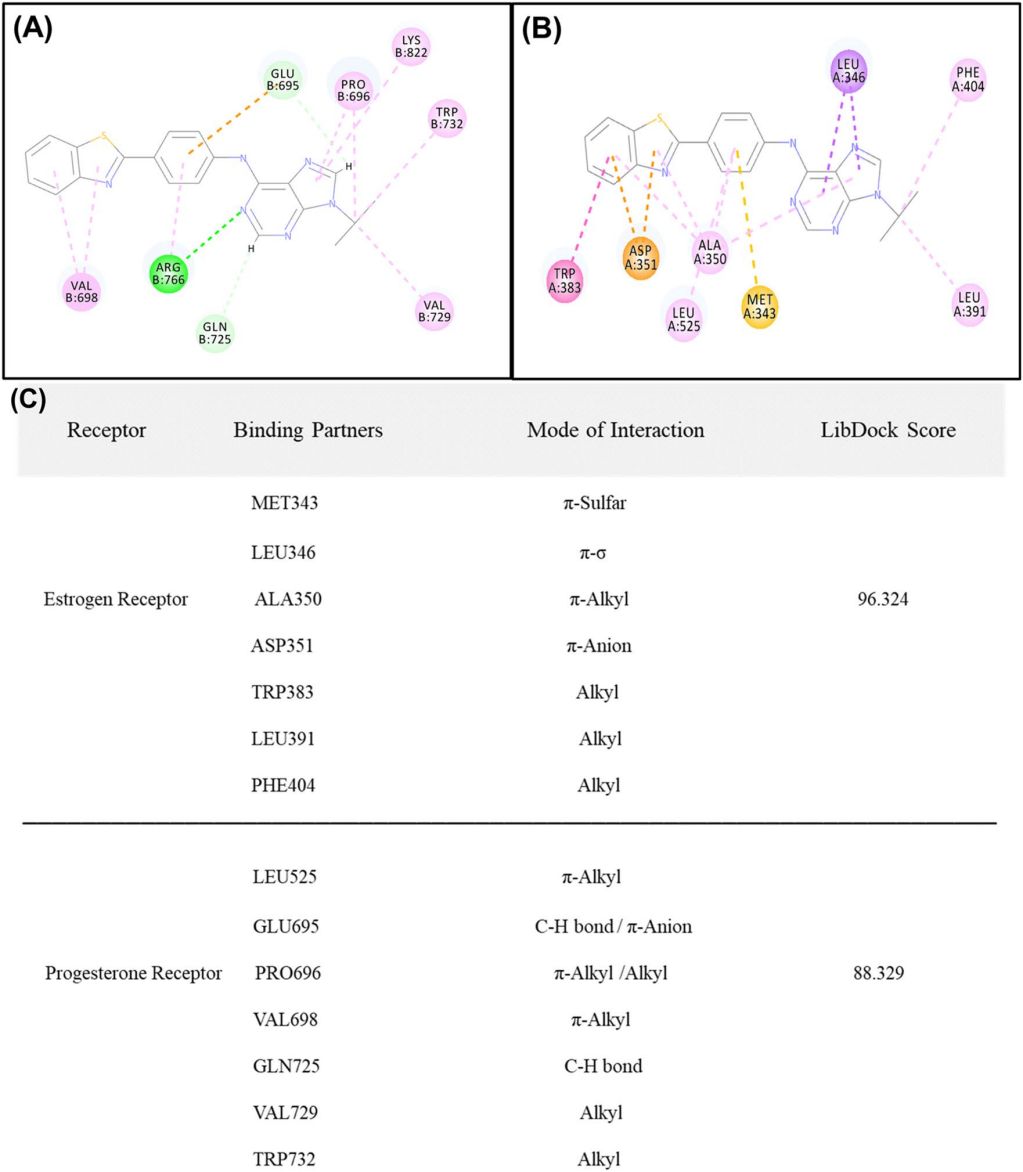
Molecular docking studies. (**A**) Benzothiazole analogue core interacting with the estrogen receptor and different amino acid interacting partners. (**B**) Benzothiazole analogue core interacting with the progesteron receptor and different amino acid interacting partners. (**C**) Table represents different binding partners and mode of interaction and binding energies of both the receptors with the benzothiazole core structure showing ER to have higher LibDock score than that of PgR.

Discovery Studio v3.5 (Accelrys, USA, 2013) was used to perform the protein and ligand preparation. LibDock program of Discovery Studio was used to determine the binding site poses that are biologically active within the active site. CharMM36 force-field was used for the energy minimization within the polar and apolar components^24^. CAESAR (Conformer Algorithm based on Energy Screening and Recursive build up) method was used for the generation of conformations keeping every other component as default ultimately to specify the structural interactions depicting in a 2D diagram of the result^25,26^. Analysis of the results showed the binding partners for both the receptors (Fig. 1A,B) and the calculated LibDock score clearly depicts (Fig. 1C) higher affinity of synthetic analogues towards the ER as compared to the PgR, thus we can continue with synthesizing the molecules and perform further validation of this hypothesis.

Furthermore, the six best fitted molecules from the 2D-QSAR study were subjected to molecular docking. The result showed CPIB, M-CPIB and Q-CPIB to possess the best LibDock score of 96.205, 95.138 and 95.002 accordingly among the six molecules (Supplementary Fig. 3). This result shows that three screened molecules among the library of fifteen molecules can be considered for further in vitro study.

### Synthesis of three screened molecules

**Figure 2:**
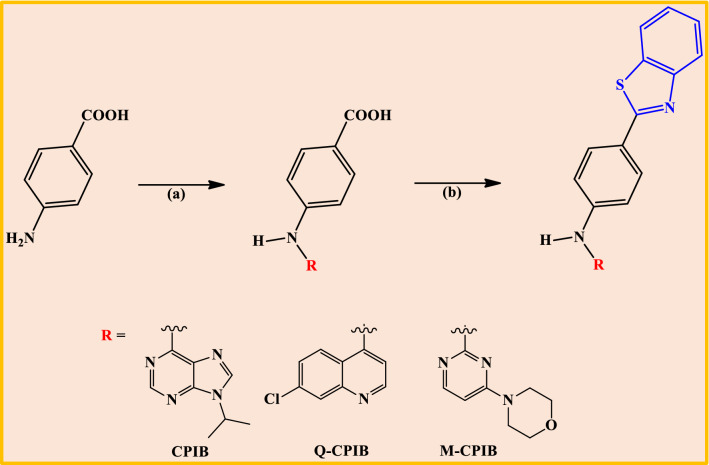
Scheme for synthesis. Reagents and Conditions: (a) R-Cl, 150 °C for 1 h in n-butanol. (b) 2-Aminothiophenol, polyphosphoric acid, 200 °C for 2 h.

**Figure 3:**
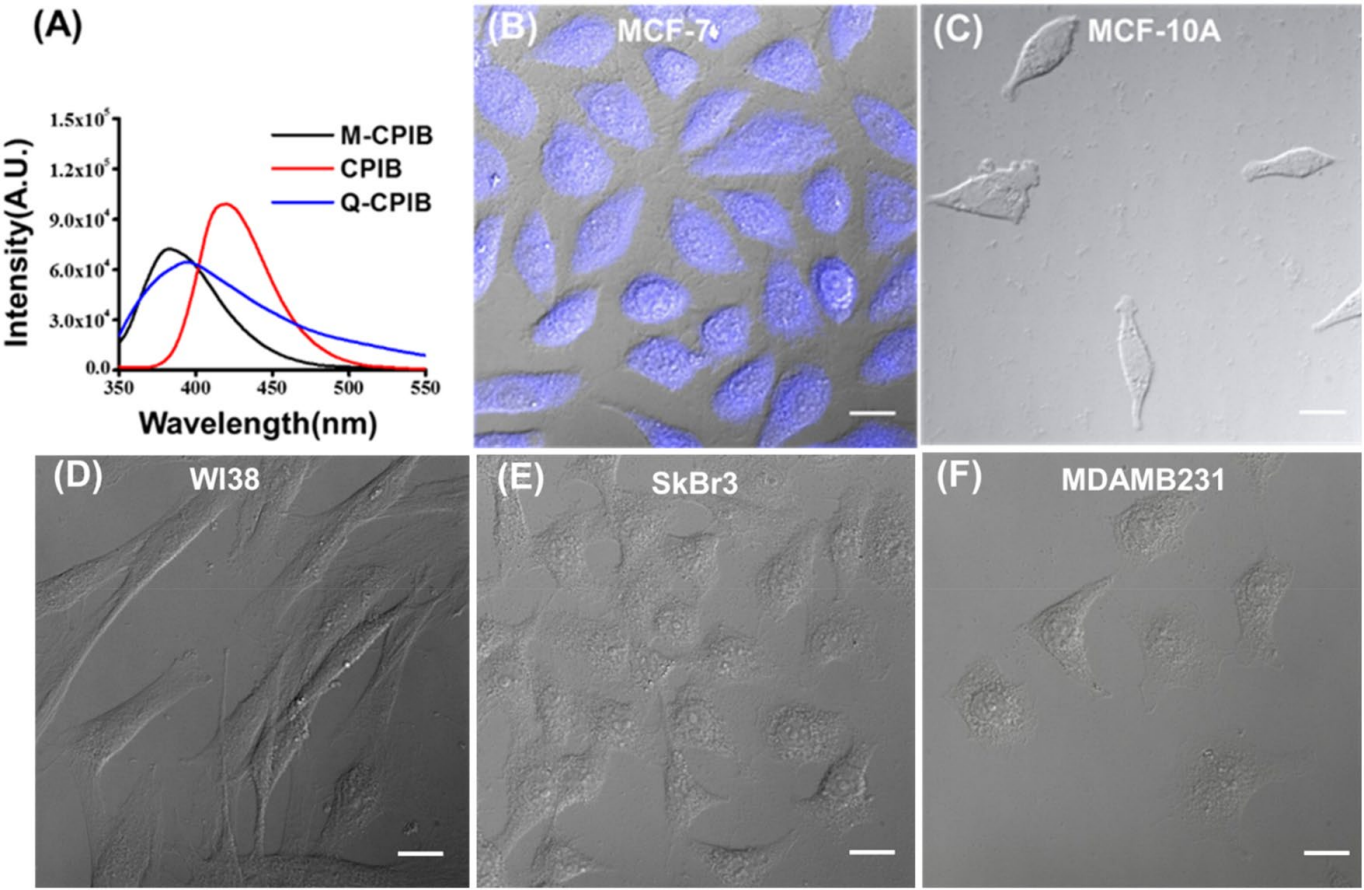
Physical characterization and cellular uptake study of CPIB. (**A**) UV–Vis absorption spectrum of CPIB, M-CPIB and Q-CPIB, CPIB having the maximum intensity among three. (**B**) DIC and fluorescence-merged image of MCF-7 cells (ER positive cells) after treatment of CPIB showed blue fluorescence of MCF-7 cells. (**C**–**F**) ER negative cell lines showing no blue fluorescence, thus clearly indicating the CPIB target ER of breast cancer cells and emits blue fluorescence. Scale bars correspond to 20 μm.

We have designed and synthesized benzothiazole based compound (CPIB, Q-CPIB and M-CPIB) and well characterized by ESI-mass spectrometry, 1H-NMR, 13C-NMR, (Fig. 2 and Supplementary Figs. 4–12). Next, we have checked the UV–Vis and fluorescence of these compounds. From the spectral analysis, we found that there were spectral responses in the region of 290–360 nm with a maxima around 335 nm by UV–Vis spectrometer (Fig. 3A). Again, we observed that there was an emission range of 390 to 485 nm when the solution of CPIB was excited at 335 nm (Fig. 3A).

### Monitoring cellular uptake using fluorescence microscope

Interestingly, all the synthesized benzothiazole derivatives showed fluorescence that motivated us to investigate the cellular uptake study using fluorescence microscopic imaging technique. Since, we have designed our molecules targeting estrogen receptor in breast cancer cells and above molecular docking result show encouraging results, we performed the cellular imaging experiment in different cell lines including ER positive and ER negative cell lines namely MCF-7 (a luminal-like cell line representing estrogen receptor positive breast cancer), MCF-10A (normal epithelial mammary gland of breast, ER negative), SkBr3 (a HER2-like cell line representing HER2 overexpressing breast cancer, ER-negative, PgR-negative, MDAMB231 (a basal-like cell line representing triple-negative breast cancer) and WI38 (normal human fibroblasts, ER-negative and PgR-negative). A varied concentration of benzothiazole derivatives were checked starting from 5 μM and going down each time half dilution upto 1 nM. Surprisingly, we found that compound CPIB showed fluorescence inside MCF-7 cell lines in all concentration in blue filter having excitation at 405 nm, whereas remaining cell lines did not show any fluorescence (Fig. 3B–F). From the results we have found that the lowest effective concentration to detect the MCF-7 cells is 1 nM of CPIB (Supplementary Fig. 13). Further, we have performed the retention of fluorescence upon treatment of 1 nM CPIB into the MCF-7 cells. We observed blue fluorescence of CPIB in MCF-7 cells till day 2. However, there is a decrease in blue fluorescence intensity (Supplementary Fig. 14). We also checked the availability of fluorescence in MCF-10A, which is a human breast epithelium cell line but did not find any fluorescence. The above result clearly shows that CPIB could potentially be used in controlled concentration as a molecular probe to detect ER positive breast cancer cells such as MCF-7 cells at a concentration of 1 nM dose. Finally, above results confirm that our design principle works efficiently towards development of highly specific molecular probes targeting ER positive breast cancer cells.

### Evaluation of role of CPIB in targeting microtubule using microtubule assembly assay

**Figure 4:**
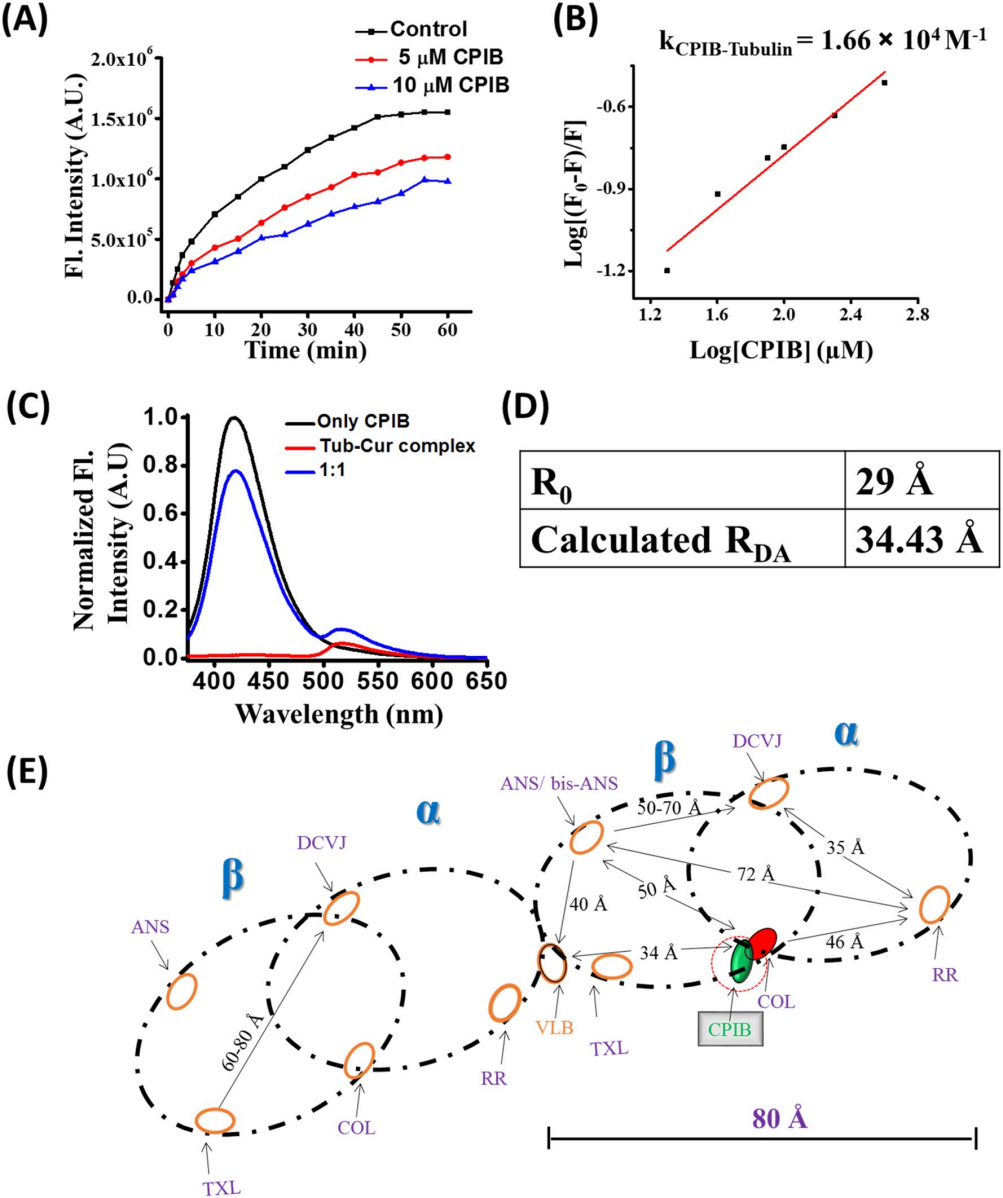
Different biophysical studies to show effect of CPIB on microtubule. (**A**) Microtubule assembly assay of CPIB of two different doses (5 μM and 10 μM). (**B**) Tryptophan fluorescence quenching experiment to determine the binding constant between tubulin and CPIB. (**C**) Förster resonance energy transfer (FRET) study: Energy transfer graph between tubulin-Cur complex and CPIB molecule. (**D**, **E**) Representation of different binding pocket and CPIB in *αβ*-tubulin heterodimer where RR (ruthenium red), ANS (8-anilino-1-naphthalenesulfonic acid ammonium salt); DCVJ (9-(dicyanovinyl) julolidine); COL, colchicine; VLB (Vinblastine).

In addition, to our design concept of development of highly specific ER positive breast cancer cell specific molecular probe, we have also considered that our designed molecules can target tubulin or microtubule as well. Therefore, we checked the interaction of CPIB molecule with tubulin by microtubule assembly assay using 4′,6-diamidino-2-phenylindole (DAPI) as a fluorescent probe. Further, the experiment was also performed using different doses of the molecule. Remarkably, we observed that the presence of CPIB molecule significantly lowered the fluorescence intensity of DAPI in a dose dependent manner, which indicates that the CPIB has potential to depolymerize the microtubule or inhibit the polymerization of microtubule (Fig. 4A).

### Determination of binding affinity of CPIB with tubulin using tryptophan fluorescence quenching assay

Microtubule assembly assay reveals that CPIB interacts and inhibits the polymerization of tubulin. Next, we tried to calculate the binding affinity of the CPIB with tubulin by tryptophan fluorescence quenching assay. The results of this experiment suggested that the binding constant of CPIB is 1.66 × 10^4^ M^−1^ (Fig. 4B). The data clearly indicates that CPIB strongly interacts with tubulin.

### Determination of binding pocket of CPIB at tubulin using FRET assay and molecular docking study

Further, we performed the Förster resonance energy transfer (FRET) experiment to get the binding location of CPIB in tubulin. Chakraborti et al., reported that curcumin binds at the pocket away from 32 Å from the colchicine binding site of tubulin^27^. Thus, we used tubulin-curcumin complex and CPIB as FRET pair. The Förster distance (R_0_) between tubulin-curcumin complex and CPIB is 29 ± 1 Å. The calculated distance between tubulin-curcumin complex and CPIB is 34.43 Å (Fig. 4C–E). Calculated distance (R_DA_) 34.43 Å indicating CPIB compounds binding away from the curcumin binding pocket with a distance of R_DA_. Moreover, we performed molecular blind docking to validate the FRET experiment data. Blind docking was performed taking CPIB as ligand and tubulin (PDB ID: 1Z2b) as a receptor. Docking results revealed that CPIB binds with tubulin at colchicine binding site with different hydrogen bonding and hydrophobic interactions with a binding affinity − 8.8 kcal/mol (Supplementary Fig. 15).

### Checking the expression of ER in different cell line through western blot

**Figure 5:**
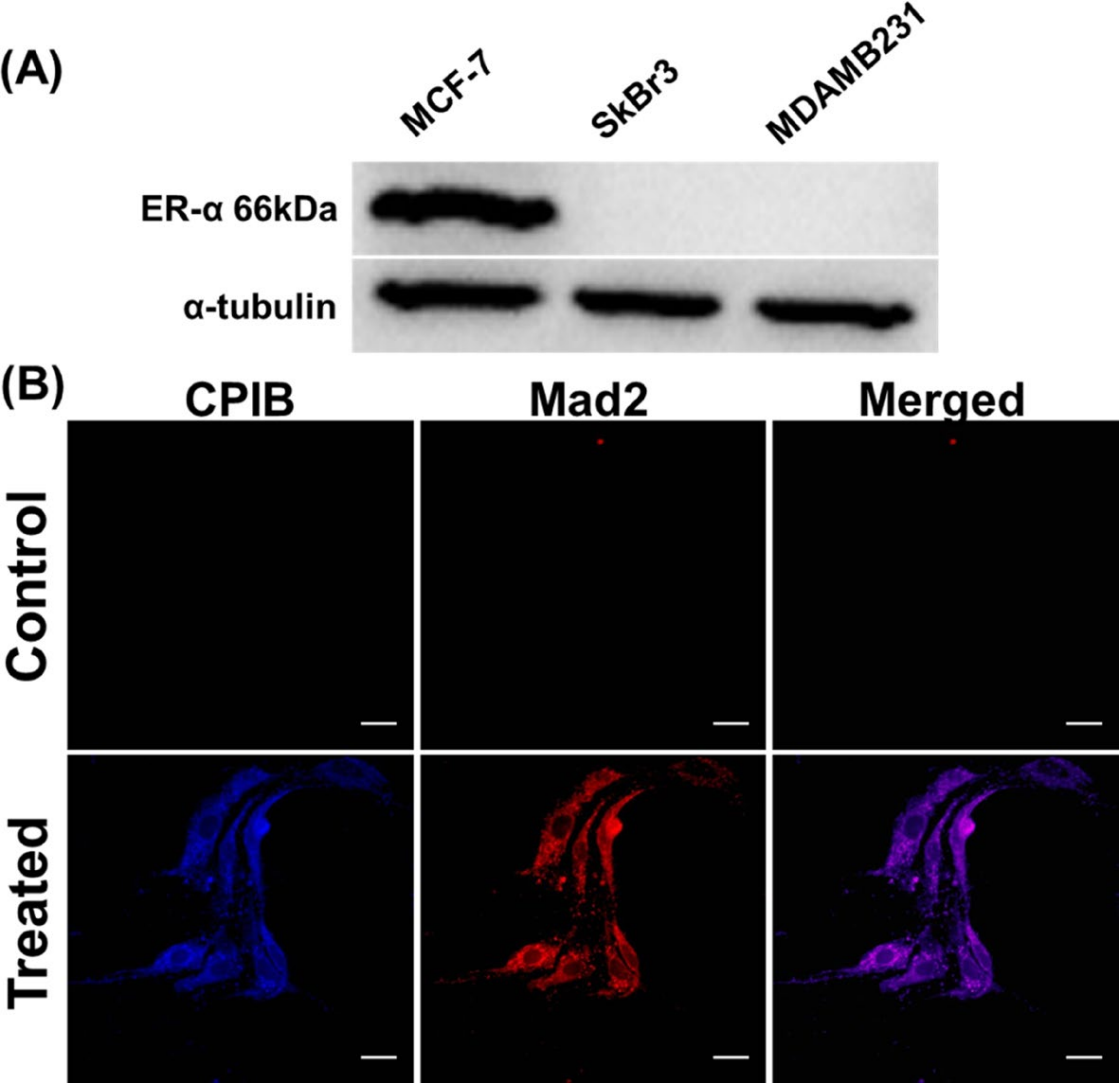
Immunoblotting of estrogen receptor α and immunocytochemistry of MAD2 expression. (**A**) Immunoblotting analysis of cell lysates collected from MCF-7, SkBr3 and MDAMB231 cells. Expression of ER-α was assayed in these cell lines whereas α-tubulin was used for loading control. Original blots/gels are presented in Supplementary Fig. 16. (**B**) Expression of MAD2 is higher after treatment of CPIB whereas control treatment shows no significant activation of the checkpoint protein. Scale bars correspond to 20 μm.

As we have checked from literature that MCF-7 among the cell line we used is ER positive cell line and our ER targeted designed molecule also showing its effectivity towards MCF-7 cell only. We were interested in checking the expression ER in these different cell lines. Interestingly from western blotting we found the expression of ER-α in only MCF-7 cell line (Fig. 5A and Supplementary Fig. 16). As we found only MCF-7 as ER positive cell line, further experiments were performed in MCF-7 cell line only.

### Determination of efficiency of CPIB in activating the mitotic checkpoint proteins Mad2 and BubR1

**Figure 6:**
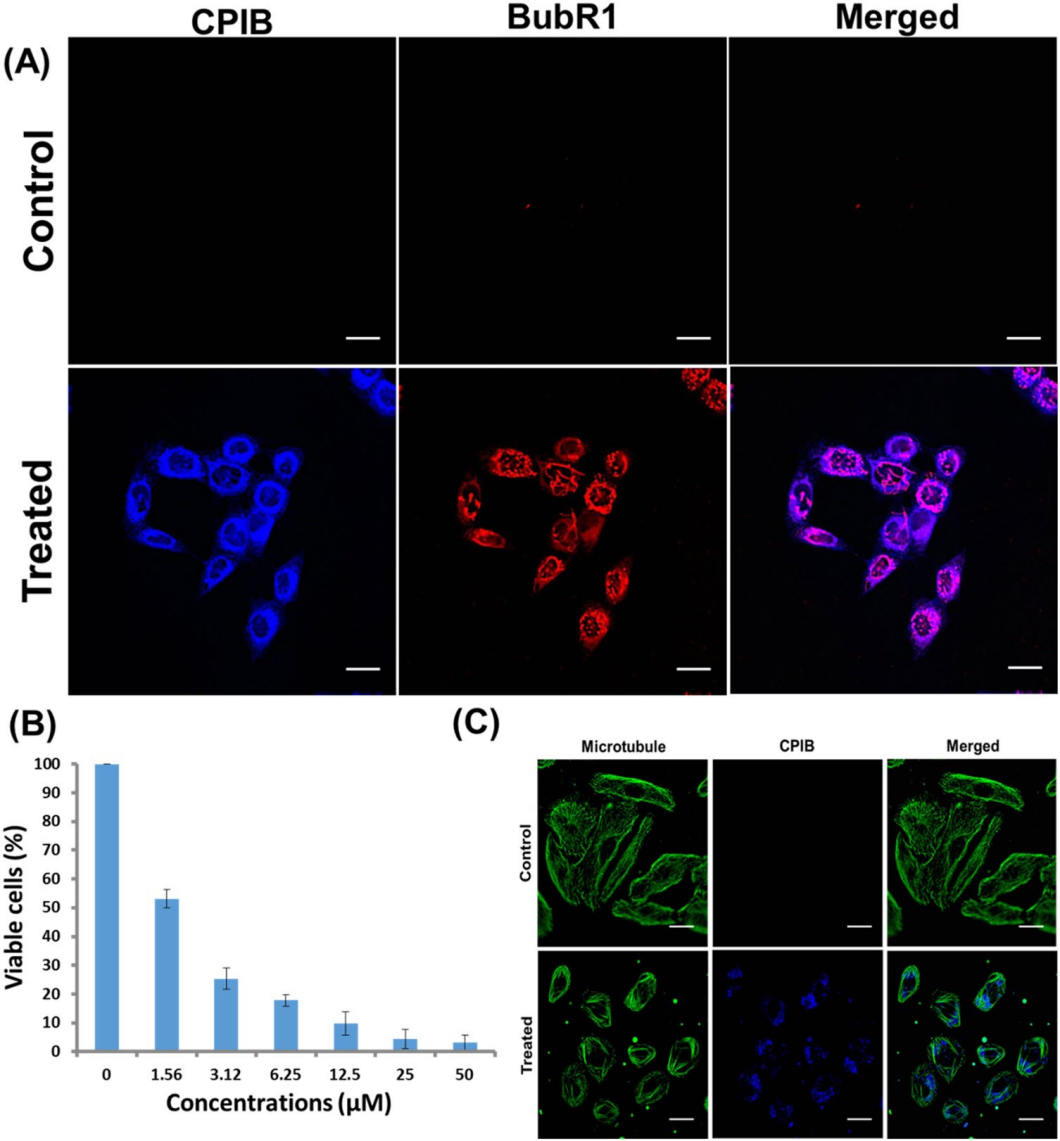
Cytotoxicity and immunocytochemistry assays of CPIB. (**A**) Expression of mitotic checkpoint protein, BubR1 after treatment of CPIB is higher compared to untreated control resulting G2/M arrest. Scale bars correspond to 20 μm. (**B**) Cytotoxicity (MTT) assay of CPIB on MCF-7 cell line showing dose dependent cytotoxicity against the breast cancer cell line. (**C**) Effect of CPIB on microtubule network, disrupting the microtubule network into a bundle shaped form as compared to the control cells. Scale bars correspond to 20 μm.

Microtubule targeting molecules are known to activate crucial mitotic check point proteins such as Mad2 and BubR1, which inhibit metaphase to anaphase transition by localizing kinetochore of chromosome resulting in G2/M arrest^28^. Initially, we have assayed the Mad2 and BubR1 proteins expression in MCF-7 after treatment with 5 μM CPIB treated and untreated cells. To investigate the activation of Mad2 and BubR1 immunocytochemistry was performed and intracellular localization was further verified. Significant higher expression level of Mad2 activation in nucleus of MCF-7 cells after treatment with 5 μM of CPIB was observed compared to untreated cells (Fig. 5B and Supplementary Fig. 17). Similarly, microscopic image revealed significant BubR1 activation in nucleus of MCF-7 cells after treatment with 5 μM of CPIB compared to control cells (Fig. 6A and Supplementary Fig. 18).

### Evaluation of cell viability of CPIB

Conventional MTT assay was used in order to check the cell viability of our novel synthesized benzothiazole based compounds. MCF-7 cells were treated with various concentrations of CPIB, Q-CPIB and M-CPIB. From the result, we found CPIB having a remarkably higher cytotoxicity compared to other analogues (Fig. 6B). We calculated the IC50 values from the cytotoxicity data and found CPIB having an IC50 value 1.3 µM (Supplementary Fig. 19A), whereas IC50 value is 1011749 µM and 6.232 µM for Q-CPIB and M-CPIB respectively (Supplementary Fig. 19B,C). We were also curious to know whether these compounds selective towards cancer cells or having cytotoxicity towards normal cells also. We performed MTT assay of our newly synthesized compounds on MCF-10A and WI38 cell line in various concentration and found that these compounds have very less or no cytotoxic effect on non-cancerous cell lines (Supplementary Fig. 20A,B). As preliminary studies reported this far in this article shows that CPIB having potential compared to other analogue and it is precisely effective on ER positive breast cancer cells (MCF-7), we moved forward with taking CPIB as lead molecule and performed further experiments on MCF-7 cell line, whereas MCF-10A cell line was used as control or normal non-cancerous cell line.

### Effect of CPIB on microtubule network

Microtubule is one of the cytoskeleton proteins that maintains the structure and function of eukaryotic cells. As microtubule assembly assay, tryptophan fluorescence quenching assay and FRET experiments confirms the binding of CPIB with microtubules, we were interested in visualize the effect on intracellular microtubule network. MCF-7 cells were treated for 4 h with 2.5 µM CPIB. The cells were then immune-stained with anti-α-tubulin primary antibody and Cy3.5 labelled anti IgG secondary antibody (Fig. 6C). The cell images were captured under fluorescence microscope (Olympus IX83) with a 40 × objective. Interestingly, we found that CPIB potentially disrupts microtubule network and converts microtubule network into bundle shaped form, whereas images of control cells of MCF-7 depict a healthy microtubule network (Fig. 6C). The result confirms that CPIB has a significant effect on intracellular microtubule shrinkages. Further we have performed co-localisation study of blue (CPIB in different conc. 5 μM and 1 nM) and green (microtubule network) by analyzing the cellular image data with the help of JACoP (Just Another Co-localisation Plugin) using ImageJ software. Co-localization analysis with JACoP represents the nice co-localization of CPIB with tubulin/microtubule (Supplementary Figs. 21a,b and 22a,b). Cytofluorogram between CPIB and FITC tubulin/microtubule network and Van Steensel’s cross-correlation functions (CCFs) for CPIB co-localization with FITC tubulin/microtubule shows good co-localization (Supplementary Figs. 21c,d and 22c,d). Pearson’s correlation coefficient found to be 1 and 0.898 for 5 μM and 1 nM of CPIB respectively. On the other hand p-value of co-localization analysis found to be 100% for both 5 μM and 1 nM of CPIB, which represents higher co-localization of CPIB with tubulin/microtubule (Supplementary Figs. 21e, 22e). On the contrary depolymerisation and bundle formation, signifying its anticancer potential.

### Determination of mode of cellular entry of CPIB

**Figure 7:**
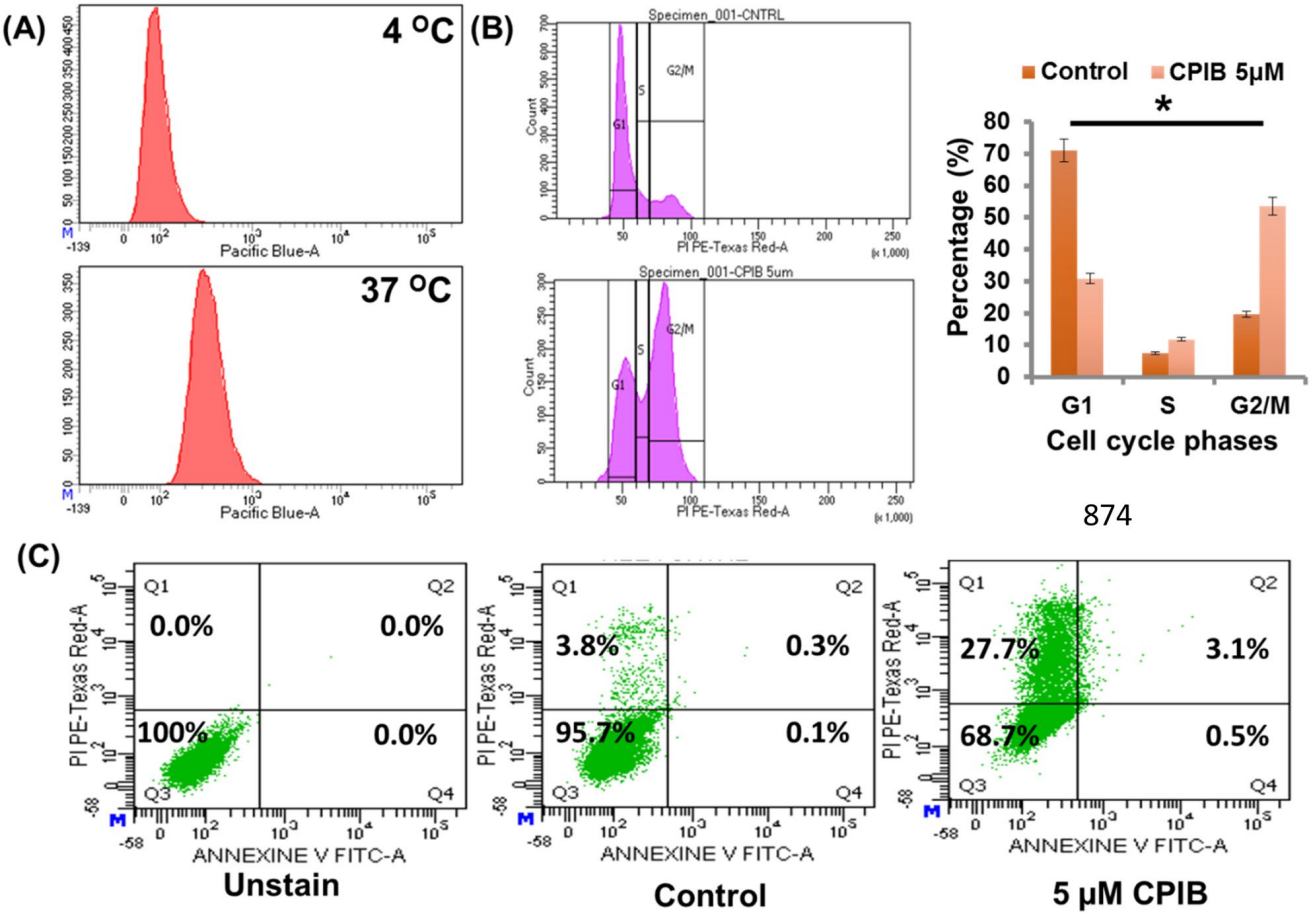
Flow cytometric assays confers apoptotic death by CPIB (**A**) Flow cytometric cell cycle analysis showing increased uptake at 37 °C compared to lower temperature at 4 °C. (**B**) Cell cycle analysis using flow cytometry after treating with CPIB (5 µM) showing arrest of cell cycle at G2/M phase. (**C**) Cellular death pathway (Apoptosis) using annexin-V and Propidium Iodide (PI) showing rapid increase in apoptotic population.

As CPIB has great effect on microtubule, it provides the insight that the compound is getting internalized by some mechanism which leads us to check the mode of internalization pathway of our novel compound CPIB. To understand mode of internalization, we have performed flow cytometric experiments as reported earlier^29^. MCF-7 cells were treated with 5 µM of CPIB. We observed significantly higher uptake of CPIB when incubated at 37 °C compared to the cells incubated at lower temperature (4 °C) (Fig. 7A). The result confirms the endocytic entry of CPIB into the ER positive breast cancer cells.

### Effect of CPIB on cell cycle

As CPIB has prominent cytotoxicity and it interacts with tubulin/microtubule network, we have further performed cell cycle analysis using flow cytometry to better understand the effect of CPIB on cell cycle. We performed the experiment in MCF-7 cell line. Briefly the cells were treated with 5 µM of CPIB for 12 h followed by fixation and PI associated cell cycle analysis in flow cytometer was performed. The result represents an increase in G2/M phase arrest (Fig. 7B). As CPIB targets the microtubule network of a cell, it generally arrests the cell cycle at G2/M phase^30^. The result from flow cytometry also confirms the CPIB as a potential microtubule binder.

### Effect of CPIB on cellular death pathway

Further, we were interested in exploring the impact of CPIB on cellular death pathway. Flow cytometric analysis was performed using annexin-V and Propidium Iodide (PI). MCF-7 cells were treated with 5 µM of CPIB. The result represents a sharp increase in apoptotic population (Fig. 7C). The reason behind may be mitotic catastrophy as CPIB having a great impact on microtubule network, the cells may be going through premature/inappropriate admittance of the cells into mitosis^31^. This result also indirectly supports the involvement of receptor mediated endocytosis process for the internalization of CPIB. On the other hand as our compound having fluorescence at DAPI/Pacific blue region we have also shown CPIB (Excitation 405 nM that is DAPI/Pacific blue region) vs annexine-V FITC and CPIB vs PE-texas red dot plot to rule out any influence of CPIB fluorescence on this experiment. Fluorescence signals came at different places at dot plot of CPIB vs annexine-V FITC and CPIB vs PE-texas red. The plots (Supplementary Fig. 23a,b) clearly showing the result of our study is not interfered by the fluorescence of CPIB in any means.

### Effect of CPIB on expression of p53 and p21

**Figure 8:**
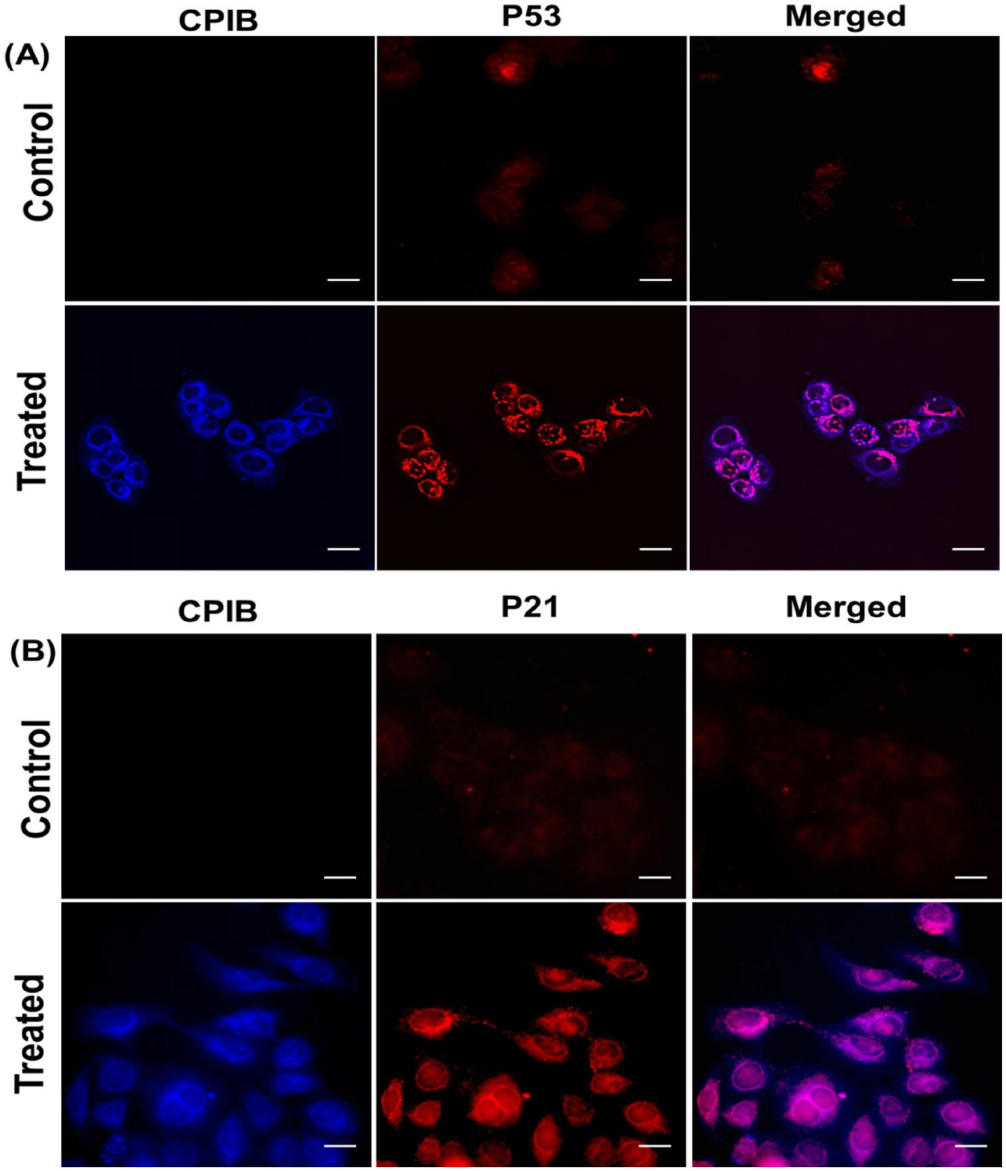
Immunocytochemistry assays confers apoptotic protein expression on treatment with CPIB (**A**) Immunocytochemistry experiment shows higher activation and localization of p53 proteins in MCF-7 cells treated with CPIB (5 μM) as compared to control signifying activation of anti-cancer pathway. Scale bars correspond to 20 μm. (**B**) Experiment shows higher activation and localization of p21 proteins in MCF-7 cells treated with CPIB as compared to control. Scale bars correspond to 20 μm.

Cancer suppressive p53 protein expression has strong correlation with stress conditions in cells such as DNA damage, oxidative stress and cytoskeleton disintegration^32^. Thus, we have analyzed the involvement of p53 in CPIB dependent cell death pathway. Here, p53 expression has been analyzed in MCF-7 cells after treatment with 5 μM CPIB using immunocytochemistry experiment. Immunocytochemistry analysis denotes nuclear localization of p53 (Fig. 8A and Supplementary Fig. 24). Next, we have studied expression of p21, which reflect p53 dependent downstream factors (Fig. 8B and Supplementary Fig. 25). We also observed its nuclear accumulation. These results signify that CPIB mediates activation of p53 and p21 anti-cancer pathways.

### Effect of CPIB on multicellular spheroidal (3D spheroid) cultures

**Figure 9:**
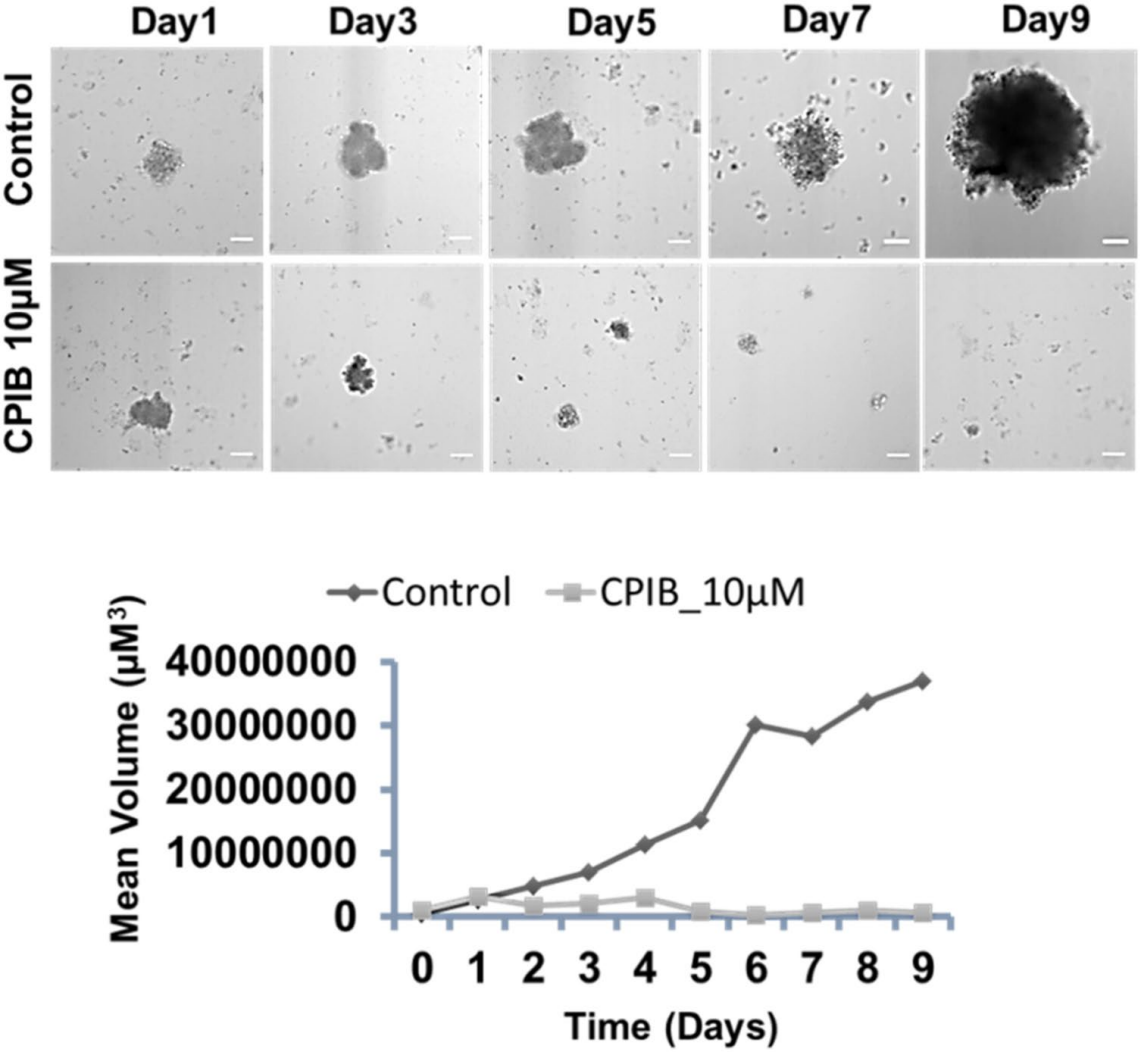
Study on tumor mimicking multicellular 3D spheroid cultures prepared from MCF-7 cell lines; Control data showing increment in the mean volume even upto 9th day, whereas CPIB treated (10 µM) spheroid shows growth inhibition (in mean volume with incubation time). Scale bars correspond to 100 μm.

So far, we have studied the effect of CPIB on cancer cell line only, we were interested in studying the efficacy of CPIB on 3D multicellular tumor spheroid model as therapeutic efficacy of any designed therapeutic molecules shows potentiality in 2D cell culture often fails to prove themselves in in vivo systems. 3D multicellular tumor model aptly mimics in vivo tumor as it has morphology, growth kinetics and micro environment similar as in vivo tumor. For our work 3D spheroid was prepared from MCF-7 cell line using liquid overlay method as CPIB has potential effect on ER positive breast cancer cell line^33^. Two sets of well grown tumor spheroids (4 days from the generation of spheroids) was taken from which one set was treated with 10 µM of CPIB and other set was kept as untreated control to compare the growth inhibition. Both the sets were imaged under inverted microscope using z-Stack, and the spheroid volume was measured up to 9 days from the day of treatment. A significant growth inhibition was observed in case of CPIB treatment in comparison to the spheroids in untreated control group (Fig. 9). The result supports the efficacy of CPIB on multicellular tumor spheroid model also. Furthermore, treatment of 10 µM CPIB in 3D spheroids of ER-negative SkBr3 cell lines resulted no growth inhibition of the treated spheroids compared to the untreated control group (Supplementary Fig. 26). The result confirms that our newly developed compound is selective towards the ER-positive cells only.

In summary, this manuscript presents successful design strategy of potential fluorescent molecular probe targeting ER positive breast cancer cells using QSAR strategy as well as molecular docking technique. Interestingly, among three screened benzothiazole-purine hybrid molecules (CPIB, M-CPIB and Q-CPIB) one molecule CPIB shows fluorescence, having excitation and emission 405 nm and 455 nm respectively. Remarkably, CPIB also showed significant potential as blue fluorescent molecular probe for ER positive breast cancer cells at a 1 nM dose. Moreover, cellular uptake study via fluorescence microscopy revealed that CPIB could specifically detect ER positive breast cancer cells starting from 1 nM concentration. Fascinatingly, we found that CPIB acts as an anticancer agent having an IC50 value 1.3 µM against ER positive MCF-7 cell line. Inspired by these results, we have identified that CPIB exerts anticancer potential by depolymerizing and bundle formation of intracellular microtubules through interacting with tubulin at curcumin binding pocket. We also observed that CPIB is highly specific to ER positive cancer cells as it does not affect normal cells. Finally, our work reports for the first time discovery of a blue fluorescent molecular probe targeted specifically to ER positive breast cancer cell up to a certain dose and above that it acts as an ER positive anti-breast cancer agent exerts its effect targeting microtubule as an intracellular molecular target.

## Methods

### Materials

Potassium carbonate, Ethylene-bis(oxyethylenenitrilo)tetraacetic acid (EGTA), 4-Piperazinediethanesulfonic acid (PIPES), Bovine serum albumin (BSA), Guanosine-5′-triphosphate sodium salt hydrate (GTP), 3-(4,5-Dimethylthiazol-2-yl)-2,5-diphenyltetrazolium bromide (MTT), Dulbecco’s Modified Eagle’s Medium (DMEM), MES, 4′,6-diamidino-2-phenylindole dihydrochloride (DAPI), Trypsin–EDTA solution, Dimethylsulfoxide for cell culture, Formaldehyde solution for molecular biology, and Propidium iodide (PI) were purchased from Sigma-Aldrich. DMSO, Potassium chloride, Ethyl acetate, Chloroform and Hexane were purchased from Spectrochem. Polyphosphoric acid, TritonX-100 and 2-[4-(2-Hydroxyethyl) piperazin-1-yl] ethanesulfonic acid (HEPES) was purchased from Sisco Research Laboratories Pvt. Ltd. (SRL). Penicillin–streptomycin and foetal bovine serum (FBS) were purchased from Invitrogen. Cover glass bottom dishes were purchased from ibidi. All the deuterated solvents were purchased from Cambridge Isotope Laboratories. All compounds were used without further purification.

### Synthesis of CPIB, Q-CPIB and M-CPIB

In first step, the chlorine of 6-chloro-9-isopropyl-9H-purine (393 mg, 2 mmol, 1 equivalent), 4-(6-Chloropyrimidin-4-yl) morpholine (398 mg, 2 mmol, 1 equivalent) and 4,7-Dichloroquinoline (396 mg, 2 mmol, 1equivalent) was displaced by 4-aminobenzoic acid (302 mg, 2.1 mmol, 1 equivalent) for 1 h in 10 mL of n-butanol. The reaction was performed at 150 °C for 1 h in a sealed pressure vessel.

In second step, equimolar amount of 2-Aminothiophenol and the appropriate substituted benzoic acids were mixed with polyphosphoric acid and heated at 200 °C for 2 h in a sealed tube vessel. Then the reaction mixture was cooled, and poured into ice-cold 30% ammonia solution. The solid product washed with water. The collected product was dried using anhydrous sodium sulphate and purified by column chromatography using mixture of ethyl acetate and hexane.

#### N-(4-(benzo[d]thiazol-2-yl) phenyl)-9-isopropyl-9H-purin-6-amine (CPIB).

^*1*^*H NMR (300 MHz, DMSO-d6) δ ppm* 1.57 (s, 3 H) 1.59 (s, 3 H) 4.80–4.88 (m, 1 H) 7.40–7.46 (m, 1 H) 7.53 (t, J = 7.68 Hz, 1 H) 8.02–8.13 (m, 4 H) 8.25 (d, J = 8.42 Hz, 2 H) 8.50 (s, 2 H) 10.29 (s, 1 H).

^*13*^*C NMR (75 MHz, DMSO-d6) δ ppm* 22.12, 46.88, 120.34, 120.57, 122.20, 122.49, 126.54, 127.71, 134.27, 140.39, 143.03, 151.46, 153.74, 167. 25; *ESI–MS* (Positive mode): Expected mass for C21H18N6S m/z is 386.47 (M), found 387.07 (M + H^+^), 409.01 (M + Na^+^).

#### N-(4-(benzo[d]thiazol-2-yl) phenyl)-7-chloroquinolin-4-amine (Q-CPIB)

^*1*^*H NMR (300 MHz, DMSO-d6) δ ppm* 7.40–7.47 (m, 1 H) 7.53 (d, J = 8.78 Hz, 3 H) 7.61 (dd, J = 8.96, 2.01 Hz, 1 H) 7.95 (d, J = 2.20 Hz, 1 H) 8.02 (d, J = 8.05 Hz, 1 H) 8.10 (d, J = 8.42 Hz, 2 H) 8.40 (d, J = 9.15 Hz, 1 H) 8.58 (d, J = 5.49 Hz, 1 H) 9.45 (s, 1 H).

^*13*^*C NMR (75 MHz, DMSO-d6) δ ppm* 119.26, 121.18, 122.55, 122.81, 124.88, 125.62, 125.83, 126.97, 128.84, 134.50, 134.59, 144.01, 152.38, 153.89, 167.34, *ESI–MS (Positive mode):* Expected mass for C22H14ClN3S m/z is 387 (M), found 387.01.

#### N-(4-(benzo[d]thiazol-2-yl) phenyl)-4-morpholinopyrimidin-2-amine (M-CPIB)

^*1*^*H NMR (300 MHz, Acetone-d6) δ ppm* 3.62–3.82 (m, 8 H), 6.31 (d, J = 6.22 Hz, 1 H), 7.38–7.45 (m, 1 H), 7.52 (td, J = 7.68,1.46 Hz, 1 H), 7.96–8.10 (m, 6 H).

^*13*^*C NMR (75 MHz, Acetone-d6) δ ppm* 45.23, 67.09, 96.70, 119.35, 119.42, 122.71, 123.49, 125.79, 126.97, 127.21, 128.92, 135.62, 145.14, 155.38, 157.18, 160.31, 163.74, 168.52. *ESI–MS (Positive mode):* Expected mass for C21H19N5OS m/z is 389.13 (M), found 390.04 (M + H^+^).

### UV–Vis and fluorescence study

A stock solution of CPIB (20 mM) was prepared in DMSO. We used 1 μM (diluted by BRB80 buffer) of CPIB for UV–Vis and fluorescence experiment by a UV–Vis spectrophotometer (Cary 60 UV–Vis Spectrophotometer, Agilent Technologies)^34^. For the fluorescence experiment in QuantaMaster Spectrofluorometer (QM-40), excitation was provided at 335 nm, and emission was collected from 350 to 550 nm. The data was calculated in OriginPro 8.5 software.

### Cell culture

MCF-7, SkBr3 and MDAMB231 cell lines were purchased from National Centre for Cell Science (NCCS) Pune, India. Cells were cultured at 37 °C and 5% CO_2_ in a humidified atmosphere using Dulbecco’s Modified Eagle Medium (DMEM) supplemented with 10% heat inactivated fetal bovine serum, penicillin (50 units/mL), streptomycin (50 μg/mL) and kanamycin sulphate (110 mg/L). MCF-10A (Normal mammary epithelial cell line) was procured from the American Type Culture Collection (ATCC) Manassas, Virginia and grown in Lonza formulated BulletKit in a humidified incubator with 5% CO_2_ and 37 °C.

### Cellular Uptake Study via Fluorescence Imaging

Different cell lines were seeded on a 35 mm cover glass bottom dish at a density of 5000 cells per dish and grown for 24 h before treatment. After 24 h cells were washed with colorless DMEM and treated with 1 nM to 2.5 μM CPIB. One disc for each group was kept untreated as control. After 4 h of treatment the cells were washed with colorless DMEM medium followed by live cell imaging using inverted fluorescence microscope Olympus IX83 with a 40 × objective in bright field and 405 nm fluorescent filters^35^. For memory test 1 nM CPIB treated MCF-7 cells were imaged under fluorescence microscope in Day 2.

### Parameter generation and preparing QSAR sets

Avogadro software was used to convert the 2D chemical structures to 3D format. The crystal structure of estrogen receptor (PDB ID: 3OS9) and progesterone receptor (PDB ID: 1E3K) were taken from RCSB Protein Data Bank (PDB) in PDB format and were prepared in Discovery Studio v3.5 (Accelrys, USA, 2013) software. Maximum contribution structure (MCS) was followed to align the training and test set^36^. Eventually, all the alignments were examined manually and the conformations with lowest energy were used. Training set and test set were selected randomly with keeping 80% in training set. Several statistical analysis were done to comprehend the QSAR model that showed a good correlation coefficient (R^2^) and cross-validation regression coefficient (q^2^).

### Molecular docking

For the docking studies, first crystal structures of target proteins were taken from RCSB database. The proteins were prepared by deleting the present inhibitor and water molecules and merging all the polar hydrogen. Next, missing atoms, conforming missing loops, and standardizing the residues were done. Upon completion, parameterization was done using Merck Molecular Forcefield 94 (MMFF94)^37^ to determine the hydrophilic and hydrophobic interactions. Top 20 poses were taken and visualized in a 2D format that depicted the amino acid partner residues from the protein target sites also.

### Tubulin purification

Tubulin was sequestered from goat brain using two cycle polymerization and depolymerization procedure as reported earlier^38^. High molarity PIPES buffer along with ATP and GTP was used as a polymerization buffer and MES based buffer was used as depolymerization buffer. The final concentration of tubulin solution was determined by measuring its absorbance at 280 nm. A stock of 20 mg/mL tubulin was prepared in BRB80 buffer and stored at − 80 °C using glycerol (10%) as a cryosolvent.

### Microtubule assembly assay

To perform the microtubule assembly assay, we have prepared a stock solution of DAPI (10 μM in buffer) which was then mixed with 100 μM tubulin and 10 mM GTP and varying doses of CPIB. We have recorded the data in the region 400 to 600 nm wavelength at the temperature of 37 °C, which excited at 355 nm^39,40^. All the collected data were plotted and analysed in OriginPro 8.5 software.

### Determination of binding affinity using tryptophan fluorescence quenching

We have calculated the binding constant of CPIB with tubulin by tryptophan fluorescence quenching experiment. To do that we have prepared different mixture having varying doses of CPIB with tubulin in BRB80. These solutions were incubated for 40 min at the temperature of 4 °C. Then, we performed the experiment and recorded the data^41^. The excitation and emission wavelengths were 295 nm and 310 nm to 400 nm respectively. The collected data were calculated by modified Stern–Volmer equation and analysed thereafter using OriginPro 8.5 software.

### Förster resonance energy transfer (FRET) study

Förster Resonance Energy Transfer Study (FRET) allows is used to measure the accurate distance between two interacting entities at angstrom distances (10–100 Å). Here, we have performed the same for the determination of binding pocket of CPIB in tubulin. We have used tubulin-curcumin complex as acceptor and CPIB molecule as donor for this experiment^42^. The efficiency of FRET was calculated by the following equation, ε_FRET_ =I_A_/(γI_D_ + I_A_); Where gamma represents the correction factor.

The Forster distance (R_0_) between tubulin-curcumin complex and CPIB was calculated to be ~ 29 Å.

Then calculated the distance (R_DA_) between tubulin-curcumin complex and CPIB using the following equation.

$$ {\text{R}}_{{{\text{DA}}}} = {\text{R}}_{o} \left( {\frac{{1 - \varepsilon_{{{\text{FRET}}}} }}{{\varepsilon_{{{\text{FRET}}}} }}} \right)^{\frac{1}{6}} $$

### Evaluation of cell viability and IC50 values

For quantitative analysis of the effect of CPIB towards MCF-7 cells was evaluated by reduction of 3-(4,5-Dimethylthiazol-2-yl)-2,5-Diphenyltetrazolium Bromide (MTT) to its insoluble formazan (MTT Assay). In Brief, MCF-7 cells were seeded at a density ~ 1 × 10^4^ cells per well in 96-well plate 24 h prior to the treatment. Then the cells were treated with different concentrations of CPIB in DMEM medium supplemented with 10% FBS for 24 h. There was no treatment in the control cells. After treatment, for all cases MTT solution (10 mg/mL) in 1 × PBS was added to each well except the background wells and incubated for further 4 h at 37 °C. Finally, the medium was discarded from each well and 1:1 mixture of DMSO-methanol was added to each well to solubilize the formazan. Absorbance of each well was recorded at 550 nm using microplate reader (Thermo Scientific Multiskan GO Microplate Spectrophotometer). Results were expressed as percentage of viability = [(A550 (treated cells)-background)/(A550 (untreated cells) − background)] × 100. IC50 values were obtained from the antilog of x-intercept of the median-effect plot^43^.

### Determination of cellular internalization pathway through flow cytometry

Mode of cellular internalization of CPIB was analysed following previously described method with minor modifications^43^. Briefly, fresh MCF-7 cells with a cell density 1 × 10^6^ were detached and collected in serum free colourless DMEM medium. The cells were then incubated at 37 °C and 4 °C differently for 40 min. Next, the cell suspensions were treated with CPIB of 2.5 μM concentration and incubated at 37 °C and 4 °C differently for further 2 h. Then the cell suspension was centrifuged to remove excess CPIB from the solution and the residue containing cell pellet was re-suspended in DMEM culture medium containing trypsin (1 mg/mL) and incubated for another 15 min. Finally, the cells were washed with serum free colourless DMEM medium and analyzed in BD LSRFortessa flow cytometer using excitation and emission filters at 405 and 455 nm respectively. BD FACSDiva software was used to analyse the data.

### Western Blot

Western blot analysis was performed following standard protocol. Briefly, MCF-7, SkBr3 and MDAMB231 cell was lysed after with RIPA buffer (10 mM Tris–HCl pH 8.0,1 mM EDTA, 0.5 mM EGTA, 1% Triton X-100,0. 1% Sodium Deoxycholate 0.1% SDS 140 mM NaCl) supplemented with protease and phosphatase inhibitors. For the protein assay we used Bradford protein assay. Briefly cell lysate protein samples and BSA standard were diluted with the Bradford protein assay reagent and absorbance values were recorded using UV-spectrophotometer at 595 nm. Protein samples were electrophoresed using 10% SDS-PAGE and transferred to PVDF membrane. Then membrane was blocked to avoid non-specific interactions and incubated with primary antibodies for overnight. Primary antibodies, such as ER-α (Estrogen Receptor α (D8H8) Rabbit mAb, CST) and anti-α-tubulin (Anti-α-Tubulin antibody, Mouse monoclonal) were used according to the manufacturer’s instruction. Next, membrane was washed and treated with HRP enzyme linked secondary antibody. Finally membrane was washed, HRP substrate added and luminescence was analysed under chemidoc.

### Cell cycle analysis by flow cytometry

The effect of CPIB on the cell cycle of MCF-7 was studied by flow cytometry. MCF-7 cells with a cell density ~ 5 × 10^5^ were treated with 5 μM of CPIB in DMEM medium containing 10% FBS for 24 h. For control experiment, there was no treatment. Then cell was trypsinized and collected by centrifugation for 3 min at 3000 rpm. All cell pellets were taken in PBS (pH 7.4) and fixed by adding cold ethanol slowly and kept at − 20 °C overnight. The final concentration of ethanol was 70% (v/v). Very next day, the cells were washed with PBS and were collected through centrifugation to remove ethanol. The cells were incubated with Propidium iodide (final concentration 100 μg/mL) and RNase A (final concentration 10 μg/mL) in PBS for 45 min at room temperature. Cell suspensions were homogenated and transferred into FACS tube and cell cycle was analysed by BD LSRFortessa flow cytometer having emission filters at 610 nm.

### Apoptosis study by flow cytometry

Pathway of cell death study was carried out for understanding the type of cell death. MCF-7 cells were seeded at a density of ~ 5 × 10^5^ cells per well in a 6-well plate one day prior to the treatment. After that the cells were treated with 5 μM of CPIB in DMEM medium containing 10% FBS for 24 h. There was no treatment in the control cell. The cells were then trypsinized and collected by centrifugation at 3000 rpm for 3 min. Then the cells were washed with PBS and collected by centrifugation and kept in dark at 37 °C for 15 min adding 100 μL solution of assay buffer containing 10 μL of Propidium iodide (PI) (stock concentration 50 μg/mL) and 2.5 μL of annexin-V (stock concentration 200 μg/mL). After that, another 400 μL of assay buffer was added to the cells and analyzed in BD LSRFortessa flow cytometer using emission filters at 530 and 610 nm respectively. BD FACSDiva software was used to analyse the data.

### Effect of CPIB on intracellular microtubule network of MCF-7 cells

The effect of CPIB on intracellular microtubule network of MCF-7 cells was performed by fluorescence microscopy. The cells were seeded on cover glass bottom dish 24 h before treatment with a cell density ~ 5 × 10^3^ cells per plate. Then the cells were treated with 2.5 µM of CPIB for 4 h in DMEM medium supplemented with 10% FBS. Then, the cells were fixed and permeabilized with 4% paraformaldehyde solution and 0.1% Triton-X-100 respectively. Then the fixed cells were treated with anti-alpha tubulin primary antibody (clone EP 1332Y Rabbit monoclonal antibody (1:300)) for 2 h followed by treatment with secondary antibody (goat pab to Rb IgG (Cy3.5) ab6941 polyclonal rabbit (1:500)) for further 2 h. Finally, the cells were washed with 1×PBS and observed under inverted fluorescence microscope (Olympus IX83) with a 40 × objective in DIC mode, 488 nm and 561 nm fluorescence channels.

### CPIB promotes activation of MAD2 and BubR1

Microtubule along with key proteins such as MAD2 and BubR1 plays important role in metaphase stage of cell division and cell cycle activation. As CPIB has a great effect on microtubule network, we were keen to know whether our compound has any role in activation of MAD2 and BubR1. For that cells were plated in glass bottom cover dish for overnight. Then, cells were treated with 2.5 µM of CPIB for 24 h. After a single wash, cells were fixed and permeabilised following earlier protocol^44^. The cells were then either immunostained with anti-MAD2 or anti-BubR1 monoclonal antibody. Red fluorophore (Cy3.5) attached secondary IgG were used in respect to anti-MAD2 or anti-BubR1. Result shows appearance of red coloured signal in CPIB treated cells than the non-treated cells. Again, the signal is appreciably localized around the nucleus. This indicates expression of MAD2 and its localization at nuclear region. Similarly, in case of BubR1 immunocytochemistry the green signal is observed around nucleus of CPIB treated cells, which is found to be absent in untreated cells. Thus, the BubR1 expression and co-localization at nucleus is resulted due to the treatment of our newly synthesized benzothiazole based compound CPIB.

### CPIB promotes elevation of p53 and p21 expression level in MCF-7 cells

Checking of expression and activation of antitumor protein p53 is essential to understand the action of anticancer chemotherapy. p53 is a gene that codes for a 53 kD protein that regulates the cell cycle and hence functions as a tumor suppression. It is very important for cells in multicellular organisms to suppress cancer. Here, the effect of CPIB on cellular p53 has been evaluated in MCF-7 cells, by using immunocytochemistry.

Briefly, MCF-7 cells were harvested overnight and treated with 5 µM of CPIB for 24 h. Then cells were washed and treated with 4% formaldehyde solution for 1 h, and 0.2% triton-X with 5% BSA for 15 min. Next, cells were washed and administrated with anti-p53 monoclonal primary antibody overnight at 4 °C followed by washing and incubation with Red fluorophore (Cy3.5) tagged anti-IgG secondary antibody. With following similar method, p21 immuno-localization was conducted with the use of anti-p21 monoclonal primary antibody. Imaging was carried out under inverted fluorescence microscope (Olympus IX83) using 40 × objective. In case of p53, we observed significant red fluorescence around the red stained nucleus of the 5 µM of CPIB treated cancer cells. However, control cell displayed no such fluorescence at 561 nm red channel. This showed the expression of p53 and furthermore nuclear localization of p53 indicates activation of this anti-tumor protein. Similar observation was observed in case of p21 expression and activation. Here again we have observed the red fluorescence around the nucleus, which was absent in control. This indicates the expression and activation of p21. As p21 is the downstream product of p53 activation, it showed that p53 is transcriptionally active to promote p21 expression.

### Generation of multicellular spheroidal (3D spheroid) cultures and treatment

Multicellular tumor spheroidal culture of cell was performed using non-adherent or liquid overlay method as described previously^33^. In brief, parental monolayer of cells were detached and pelleted. The cell pellets were then inoculated into 35 mm culture dish, previously coated with 1% agarose (weight/vol.). The inoculation concentration was maintained ~ 5 ×10^3^ cells per plate. RPMI-1640 (10% FBS, 100 μg/mL penicillin and 100 μg/mL streptomycin) culture medium was used in this process and culture dishes were incubated undisturbed at 37 °C humidified chamber with 5% CO_2_ till spheroid formation. Then morphological structures of spheroid were captured in DIC mode using an inverted microscope (Olympus IX83) equipped with EMCCD camera. Images were represented as day zero images. Then 35 mm dish were divided into different treatment group and marked accordingly. Then each plate was treated with 10 µM of CPIB. One control dish was kept. All the dishes were studied everyday under inverted microscope (Olympus IX83) using Z-stack and the volume of the spheroid was measured upto 9 days from the day of treatment. Volume of the sphere was analysed using following pre-reported formula^45^.

$$ {\text{V }} = \, 0.{5 }*{\text{ Length }}\left( {{\text{Major}}\;{\text{ Axis}}} \right) \, *{\text{ Width}}^{{2}} \left( {{\text{Minor}}\;{\text{ Axis}}} \right) $$

### Data analysis

Microscopic images were analysed using cellSens and ImageJ software. FACS data was analysed using BD FACSDiva software.

## Supplementary Information

Supplementary Figures.
