# Supplementary material for: A potent estrogen receptor and microtubule specific purine-benzothiazole-based fluorescent molecular probe induces apoptotic death of breast cancer cells
Source: Sci Rep. 2022 Jun 24;12:10772. doi: 10.1038/s41598-022-12933-8 (PMC9232585; doi:10.1038/s41598-022-12933-8)
Supplement: Supplementary file 1 — Supplementary Figures. [file 41598_2022_12933_MOESM1_ESM.docx]

A Potent Estrogen Receptor and Microtubule Specific Purine-Benzothiazole-based Fluorescent Molecular Probe Induces Apoptotic Death of Breast Cancer Cells

Surajit Barman,^#1^ Subhajit Ghosh,^#1^ Rajsekhar Roy,^2^ Varsha Gupta,^1^ Satyajit Ghosh,^2^ Surajit Ghosh^1, 2, 3 *^

1. Organic and Medicinal Chemistry and Structural Biology and Bioinformatics Division, CSIR-Indian Institute of Chemical Biology, Kolkata 700 032, West Bengal, India.

2. Department of Bioscience & Bioengineering, Indian Institute of Technology Jodhpur, Karwar, Rajasthan 342037, India.

3. Academy of Scientific and Innovative Research (AcSIR), Ghaziabad 201002, India.

^#: Equal Contribution^

**CORRESPONDING AUTHOR INFORMATION:**

Email: [sghosh@iitj.ac.in](mailto:sghosh@iitj.ac.in); sgiicb@gmail.com

Supplementary Information

**
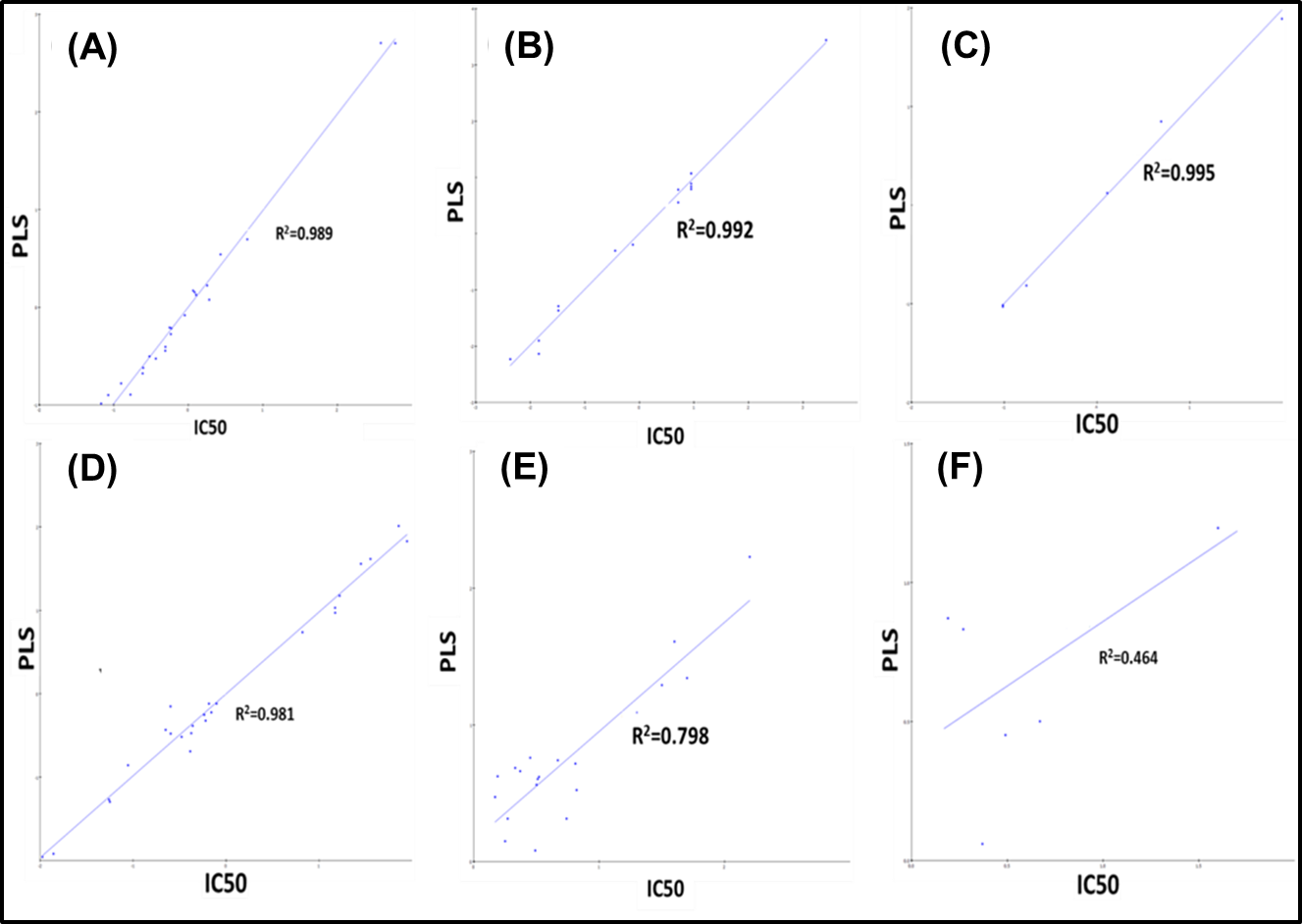
**

**Supplementary Fig 1:** (A) Partial least-square model generated keeping the IC50 value against Estrogen receptor (ER) as dependent property showing square correlation coefficient value of ≥ 0.9 as a standard. (B) Validation of the Fifteen designed molecules through 2D-QSAR model showing good square correlation coefficient value of 0.992. (C) Validation of the six designed molecules through 2D-QSAR model showing good square correlation coefficient value of 0.995. (D) Partial least-square model generated keeping the IC50 value as a standard against progesterone receptor (PR) as dependent property showing square correlation coefficient value of ≥ 0.9. (E) Validation of the fifteen designed molecules through 2D-QSAR model showing poor square correlation coefficient value of 0.798. (F) Validation of the Fifteen designed molecules through 2D-QSAR model showing poor square correlation coefficient value of 0.464.

**
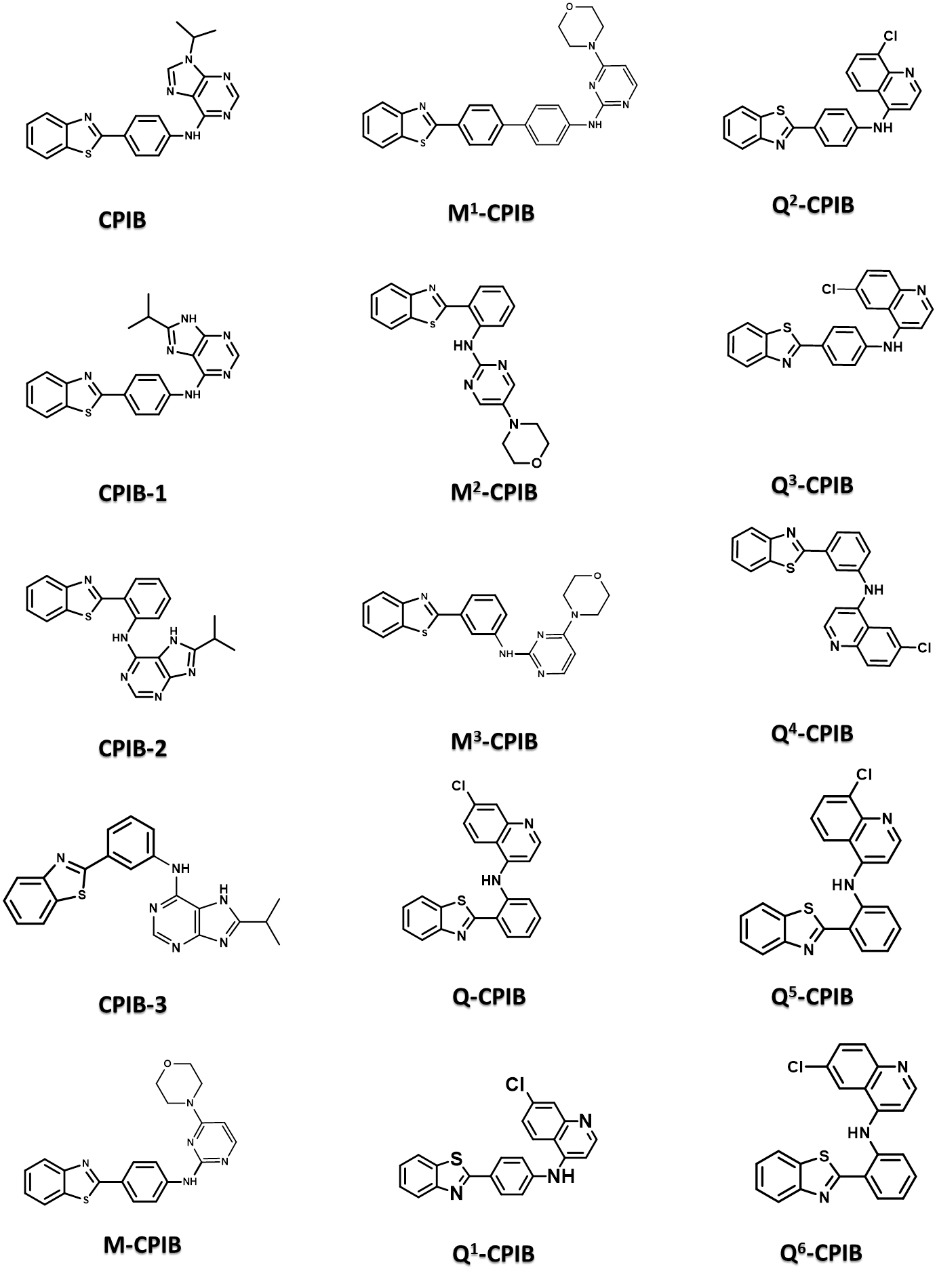
**

Supplementary Fig 2: All the 15 molecules that were designed on the basis of benzothiazole- core.


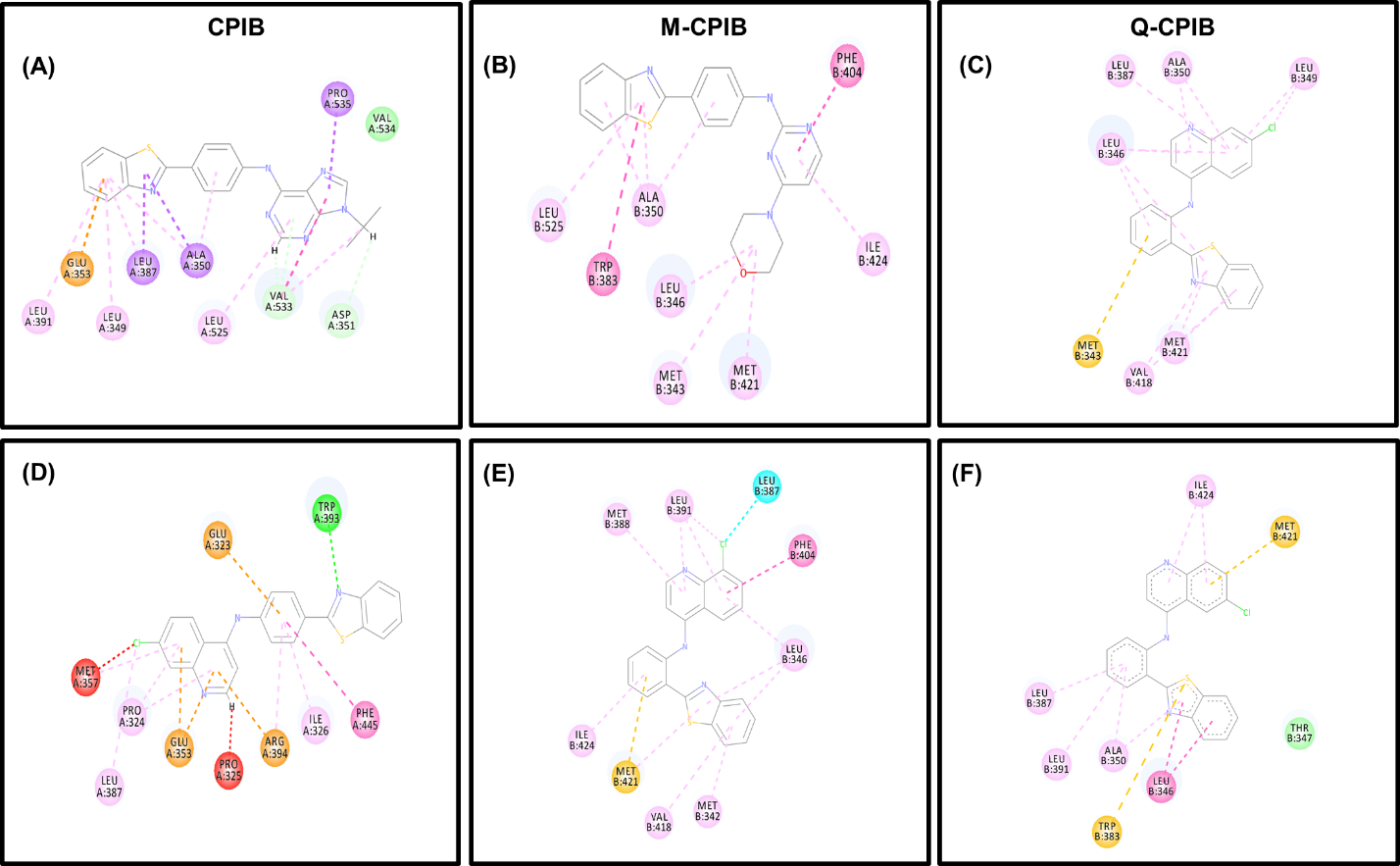


Supplementary Fig 3: (A-F) Molecular docking images and corresponding binding partners of six of the best-docked molecules. CPIB, M-CPIB and, Q-CPIB showed the best docking score among these six molecules with the LibDock score 96.205, 95.138 and 95.002 accordingly.


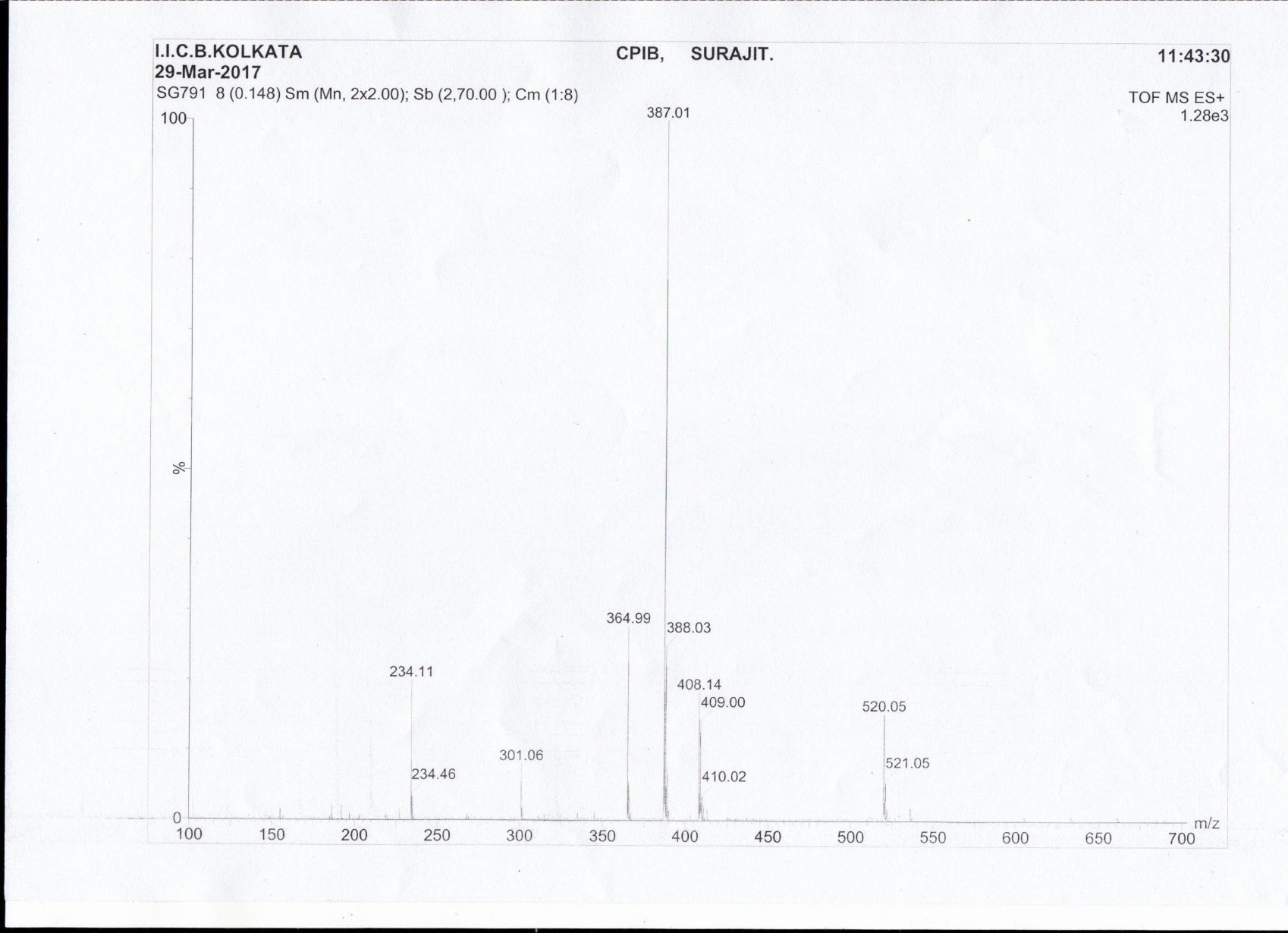


Supplementary Fig 4: ESI Mass spectrum of Q-CPIB. 387.07 Da (M), 409.01 Da (M+ Na^+^) and 520.05 Da (M+ 4K^+^).





Supplementary Fig 5: ^1^H-NMR spectrum of Q-CPIB *(300 MHz, DMSO-d_6_)*.




Supplementary Fig 6: ^13^C-NMR spectrum of Q-CPIB.


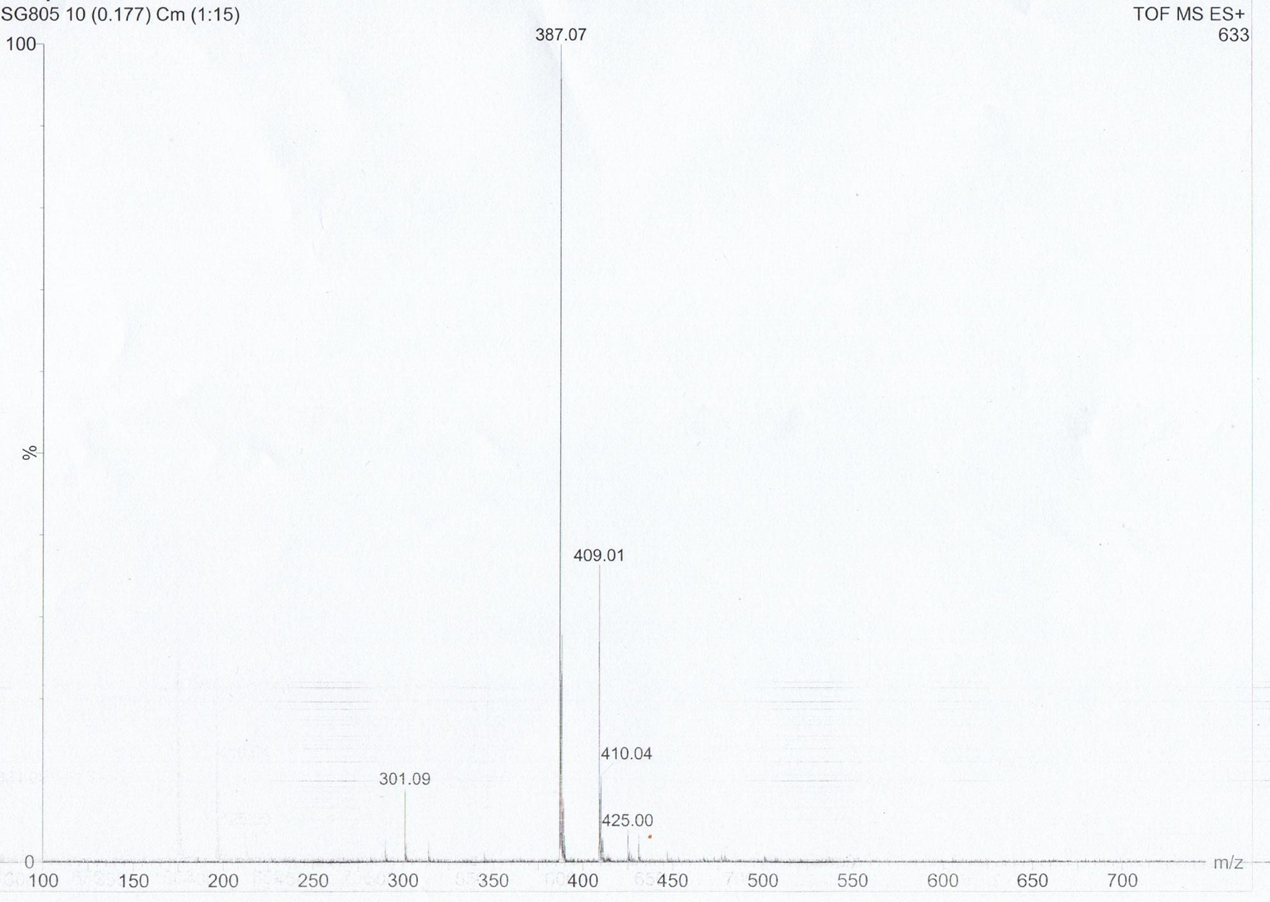


Supplementary Fig 7: ESI Mass spectrum of CPIB showing 387.07 Da (M) and 409.01 Da (M + Na^+^) peaks.

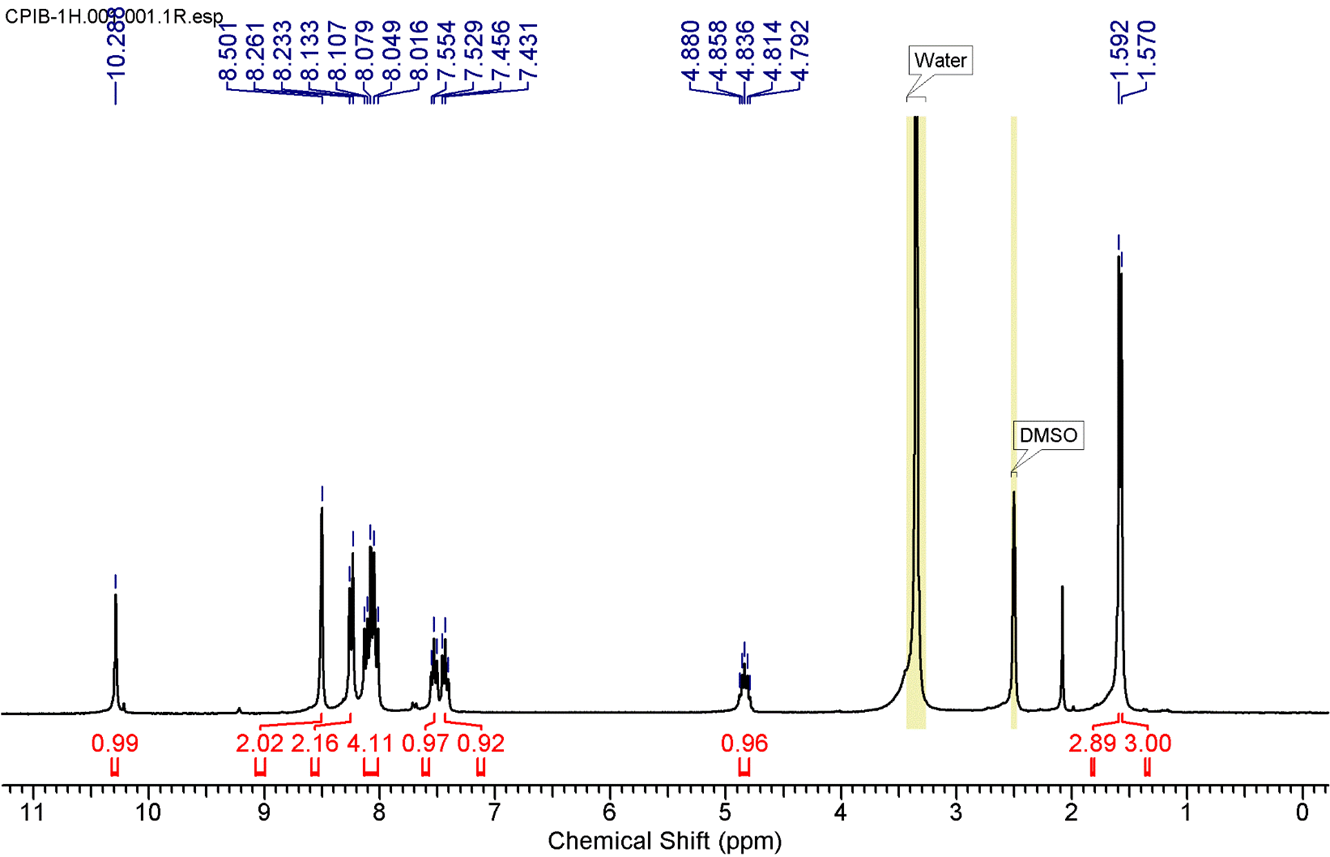
Supplementary Fig 8: ^1^H-NMR of CPIB (*300 MHz, DMSO-d6*).





Supplementary Fig 9: ^13^C- NMR spectrum of CPIB.


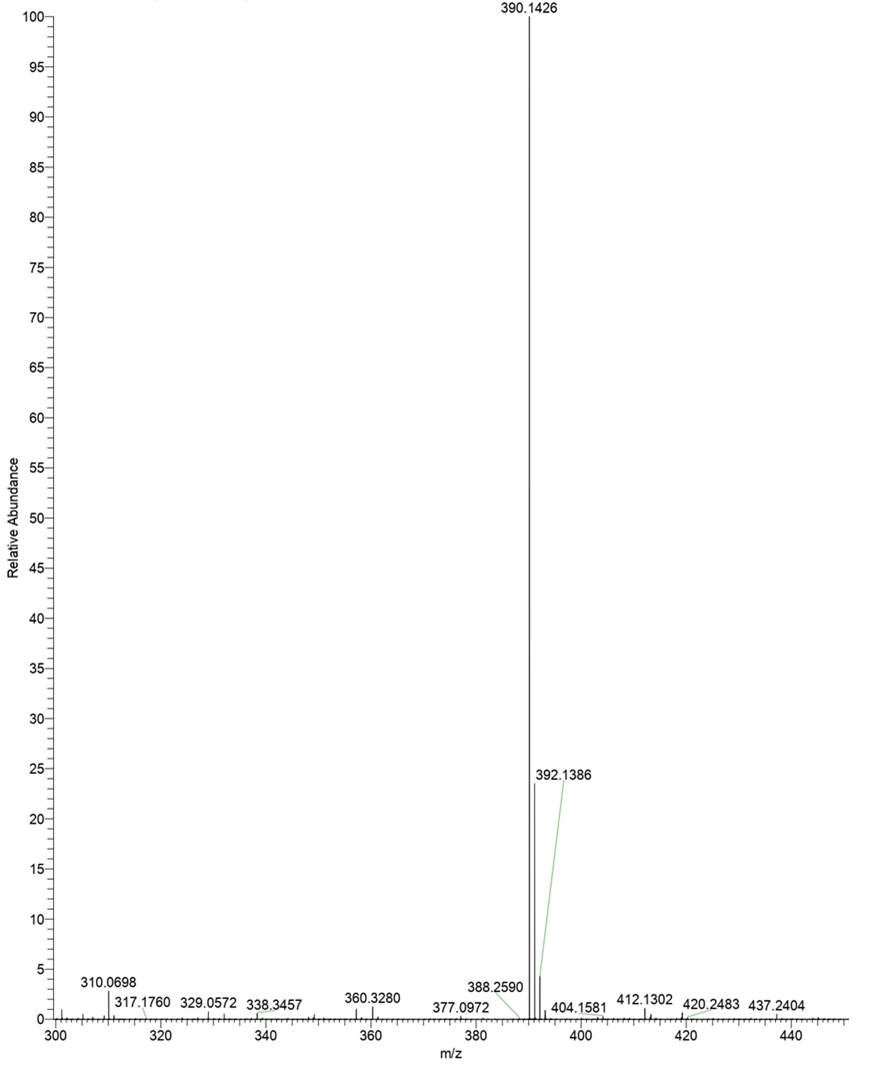

Supplementary Fig 10: Mass spectrum of M-CPIB.





Supplementary Fig 11: ^1^H-NMR spectrum of M-CPIB *(300 MHz, Acetone-d_6_)*.





Supplementary Fig 12: ^13^C-NMR spectrum of M-CPIB.


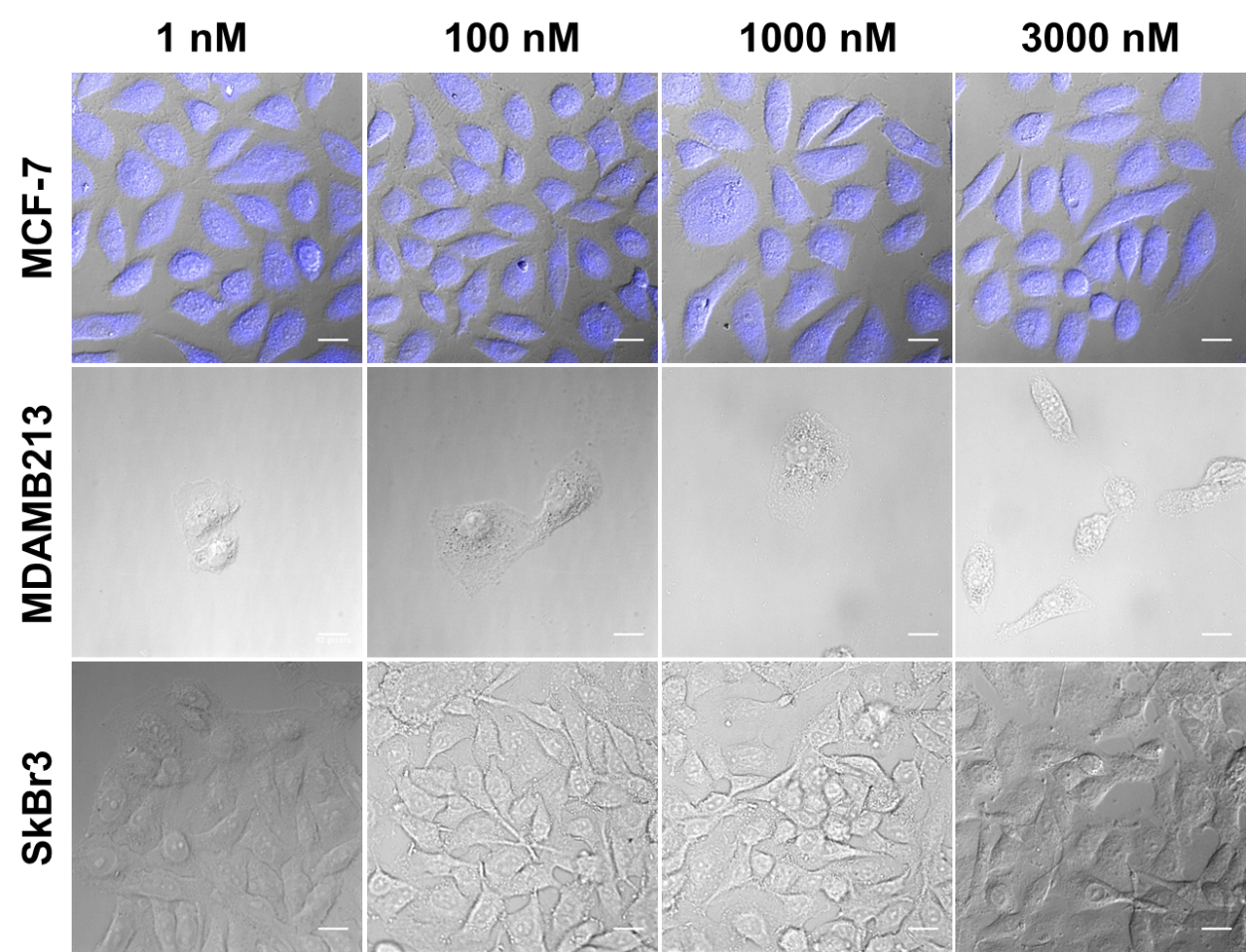


Supplementary Fig 13: Dose dependent treatment of CPIB on different cell lines. Imaging of different cell line using CPIB, result shows that CPIB only interact with ER positive MCF-7 cell line and illuminate blue fluorescence. Scale bars corresponds to 20 μm.


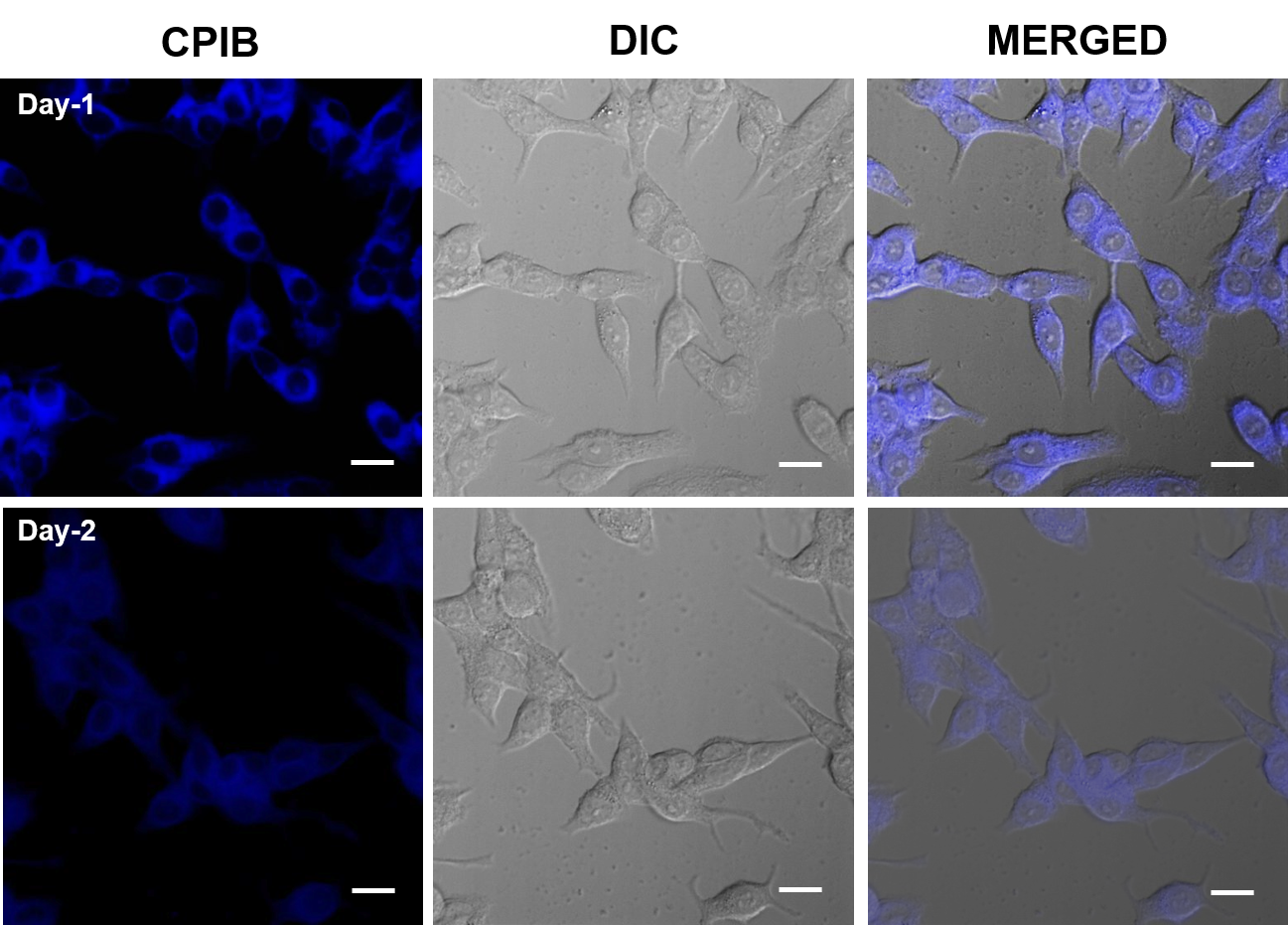


Supplementary Fig 14: Retention of blue fluorescence of 1 nM CPIB treated MCF-7 cells. Results indicate that blue fluorescence exist in cells till day 2. However, fluorescence intensity reduces compared to day 1. Scale bars correspond to 20 μm.


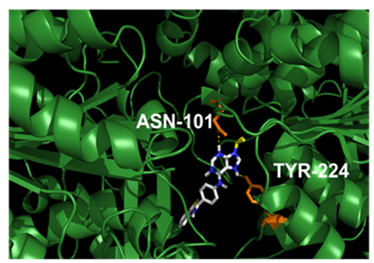


Supplementary Fig 15: Molecular docking study and binding partners of CPIB. (PDB ID of tubulin: 1Z2b)


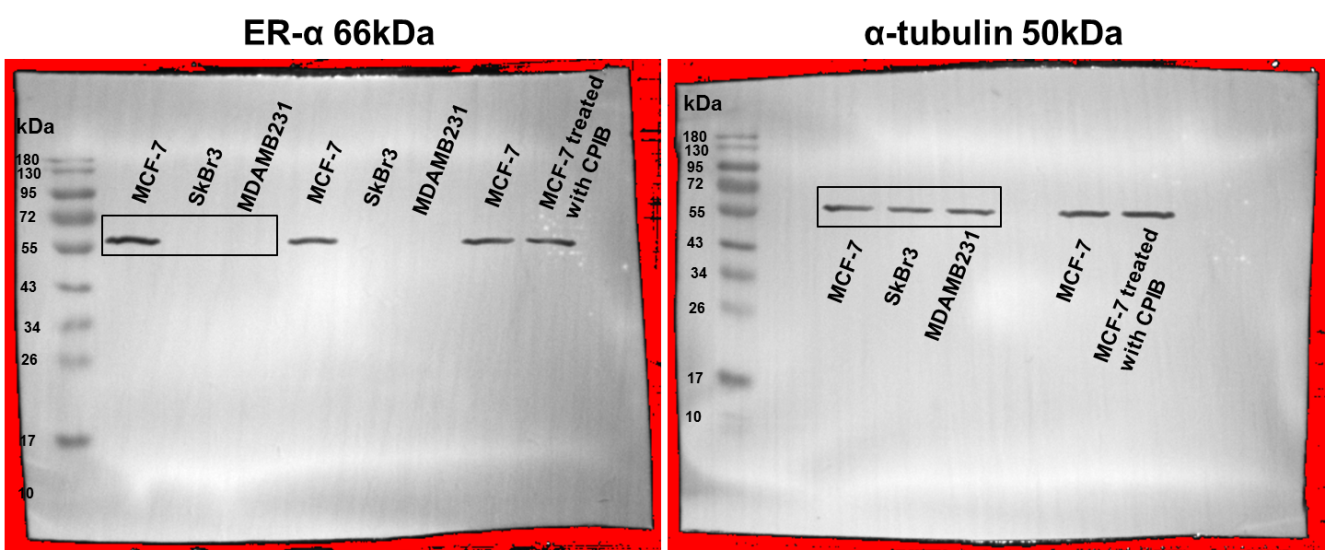


Supplementary Fig 16: Immunoblotting analysis of cell lysates collected from MCF-7, SkBr3 and MDAMB-231 cells. Expression of ER-α was assayed in these cell lines whereas α-tubulin was used for loading control.


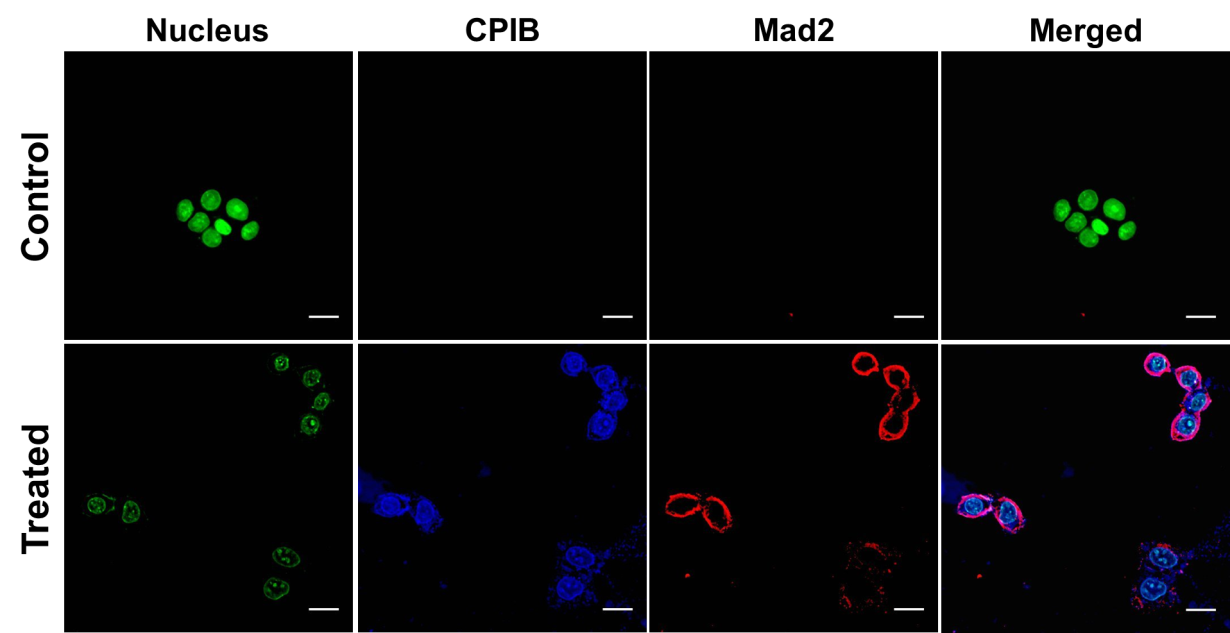


Supplementary Fig 17: Expression of MAD2 is higher after treatment of CPIB whereas control treatment shows no significant activation of the checkpoint protein. Scale bars correspond to 20 μm.


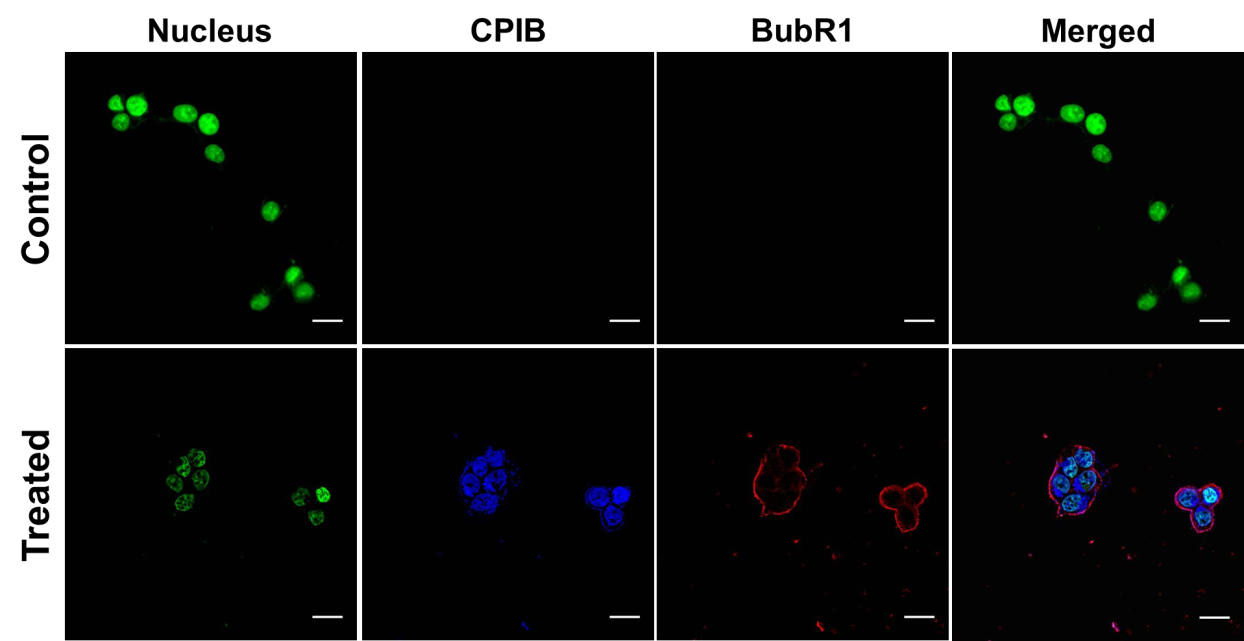


Supplementary Fig 18: Expression of mitotic checkpoint protein, BubR1 after treatment of CPIB is higher compared to untreated control resulting G2/M arrest. Scale bars correspond to 20 μm.


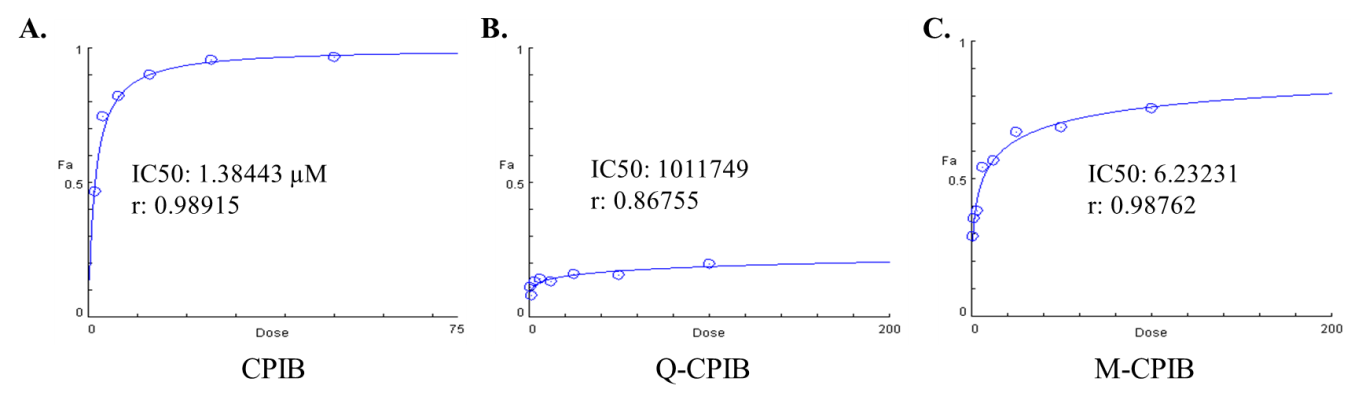


Supplementary Fig 19: IC50 values from the cytotoxicity data on MCF-7. (A) CPIB having an IC50 value 1.3 µM. (B-C) Whereas Q-CPIB and M-CPIB having an IC50 value 1011749 µM and 6.232 µM respectively.


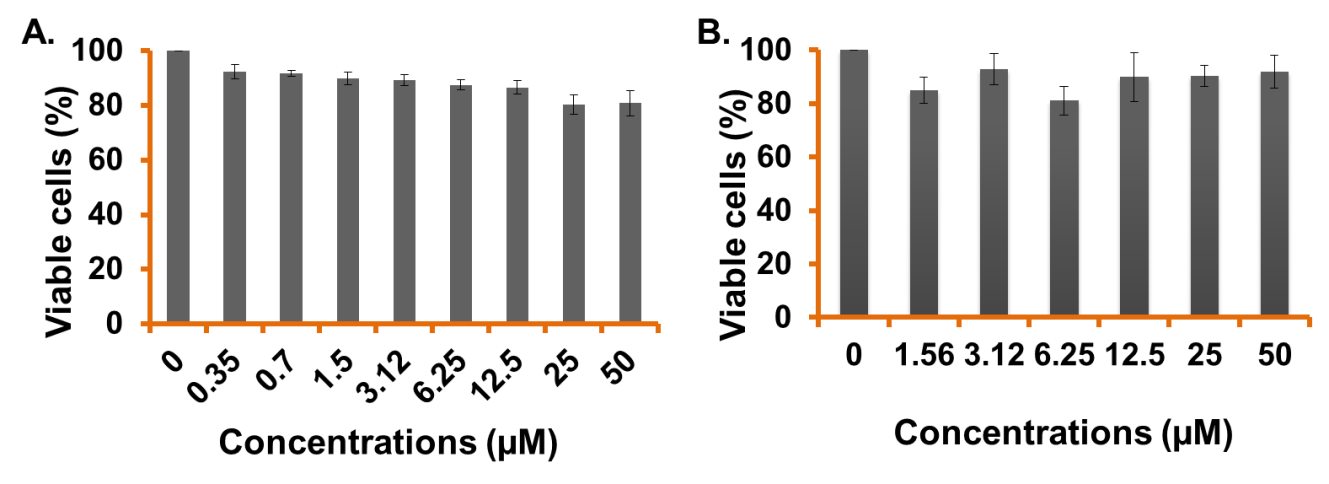


Supplementary Fig 20: MTT assay of CPIB on MCF-10A (A) and WI38 (B) cell line in various concentrations. Result showing insignificant or no cytotoxic effect on the cell lines, thus showing the importance of ER interaction.


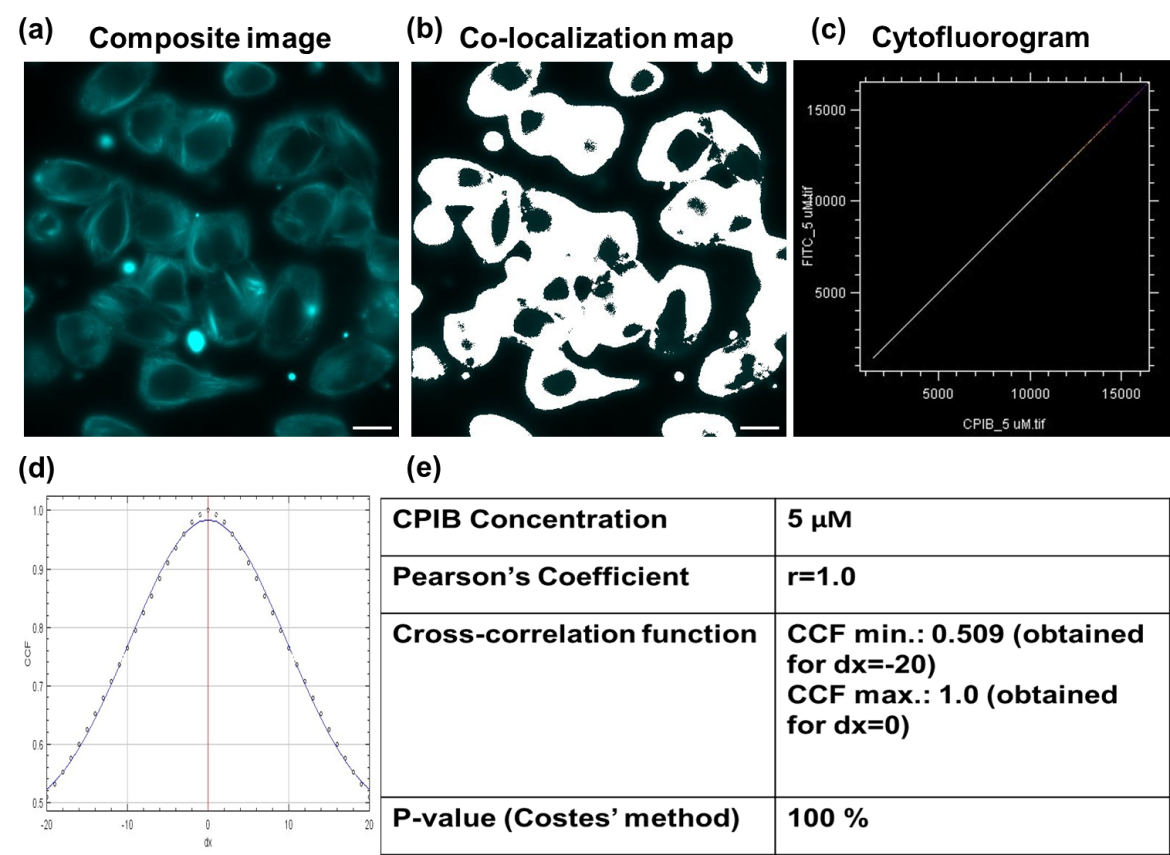


Supplementary Fig 21: Co-localization analysis with JACoP MCF-7 cells treated with 5 μM of CPIB, image randomization of CPIB (Blue) and FITC (Green microtubule) channel generates composite image (a) and co-localisation map (b) shown as white overlay on merge of the blue and green channel. Scale bar corresponds to 20 μm. The result represents the nice co-localization of CPIB with tubulin/microtubule. (c) Cytofluorogram between CPIB and FITC tubulin/microtubule network shows good co-localization. (d) Curve represents Van Steensel’s cross correlation functions (CCFs) for CPIB co-localization with FITC tubulin/microtubule. (e) Table represents Pearson’s coefficient, CCF and p-value of co-localization analysis represents higher co-localization of CPIB with tubulin/microtubule.


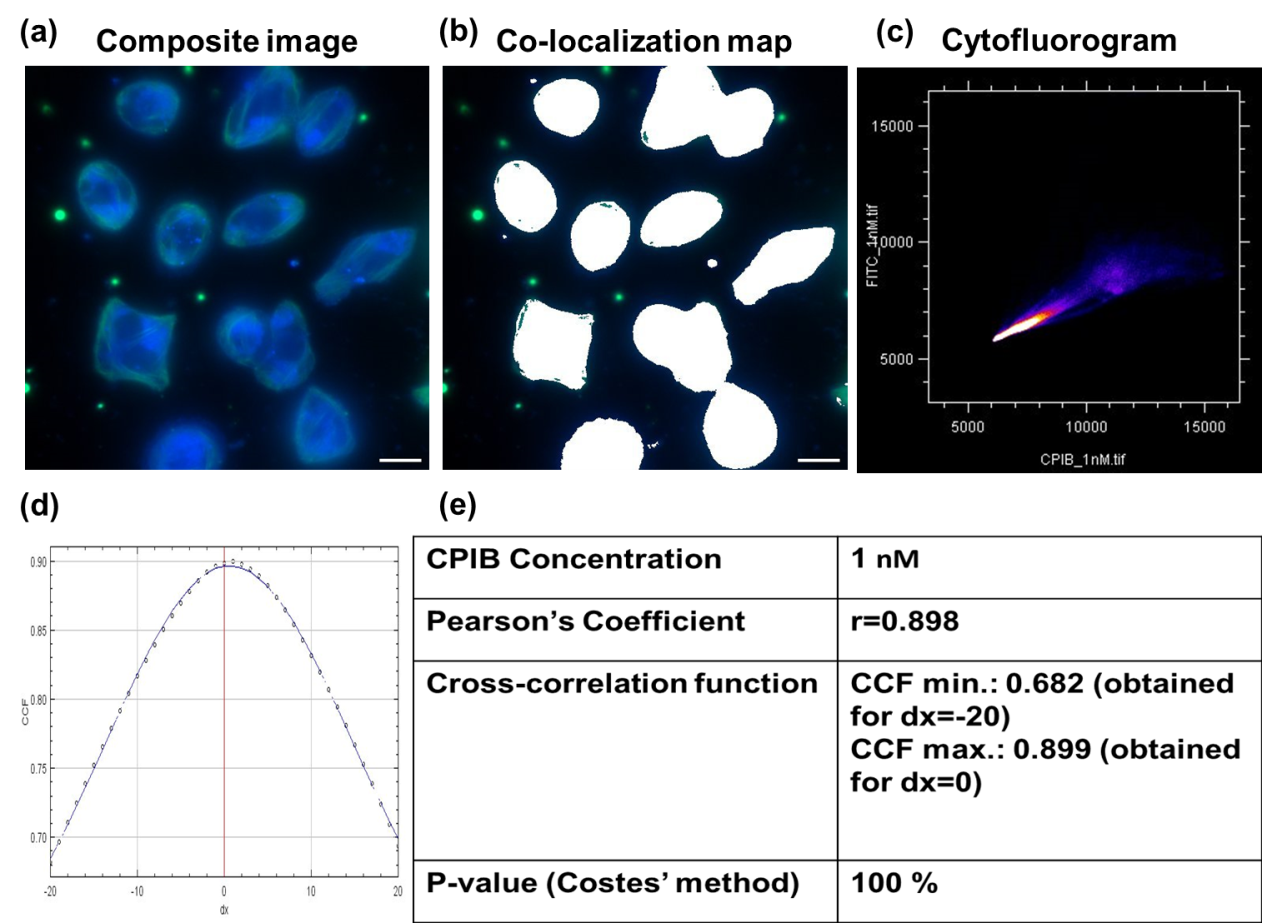


Supplementary Fig 22: Co-localization analysis with JACoP MCF-7 cells treated with 1 nM of CPIB, image randomization of CPIB (Blue) and FITC (Green microtubule) channel generates composite image (a) and co-localisation map (b) shown as white overlay on merge of the blue and green channel. Scale bar corresponds to 20 μm. The result represents the nice co-localization of CPIB with tubulin/microtubule. (c) Cytofluorogram between CPIB and FITC tubulin/microtubule network shows good co-localization. (d) Curve represents Van Steensel’s cross correlation functions (CCFs) for CPIB co-localization with FITC tubulin/microtubule. (e) Table represents Pearson’s coefficient, CCF and p-value of co-localization analysis represents higher co-localization of CPIB with tubulin/microtubule.


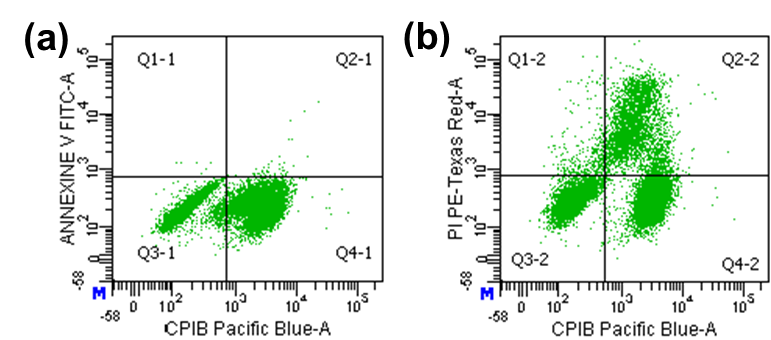


Supplementary Fig 23: Dot plot (a) CPIB (DAPI/Pacific Blue-A) vs Annexine-V FITC-A (Q1-1: 0.0%, Q2-1: 0.3%, Q3-1: 33.6% and Q4-1: 66.1%) and (b) CPIB Pacific Blue-A vs PI PE-Texas Red-A dot plot (Q1-2: 202%, Q2-2: 25.1%, Q3-2: 29.7% and Q4-2: 43.0%).


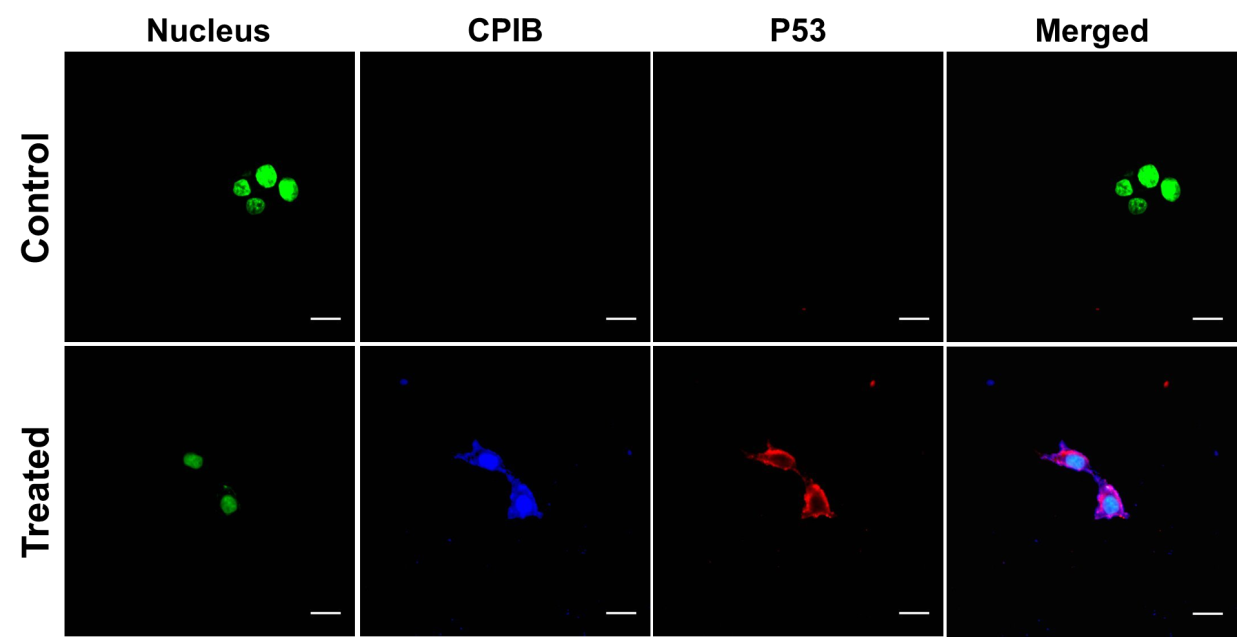


Supplementary Fig 24: Immunocytochemistry experiment shows higher activation and localization of p53 proteins in MCF-7 cells treated with CPIB (5 μM) as compared to control signifying activation of anti-cancer pathway. Scale bars correspond to 20 μm.


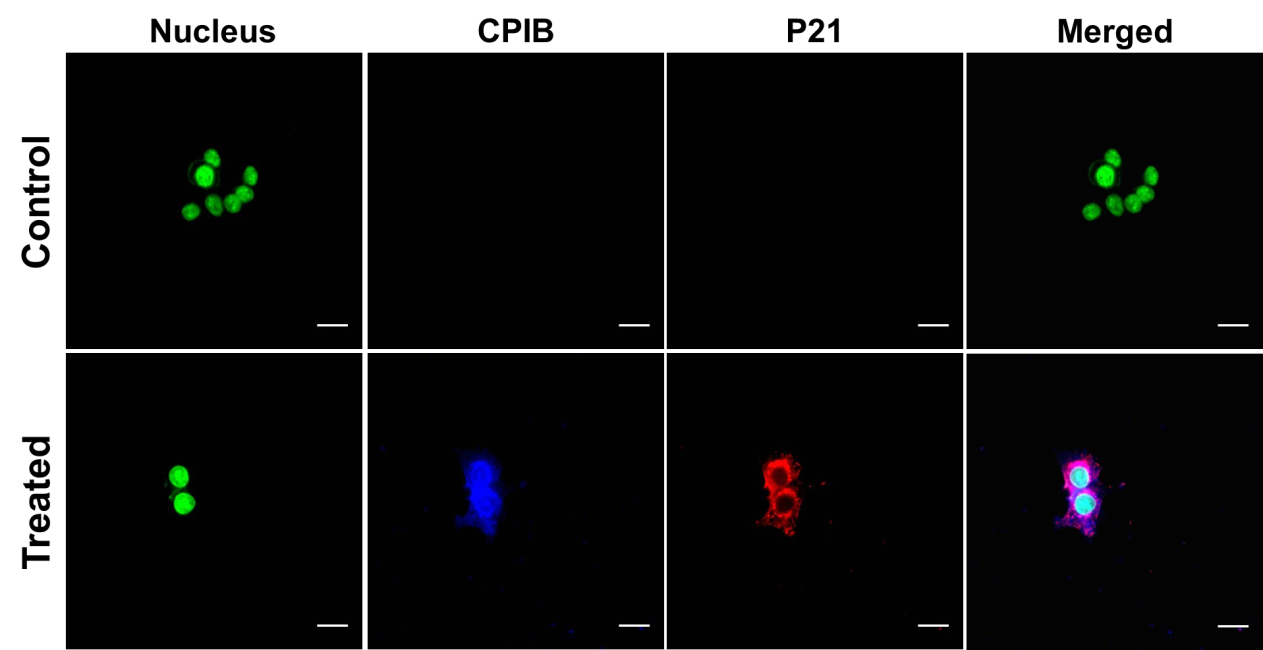


Supplementary Fig 25: Experiment shows higher activation and localization of p21 proteins in MCF-7 cells treated with CPIB as compared to control. Scale bars correspond to 20 μm.


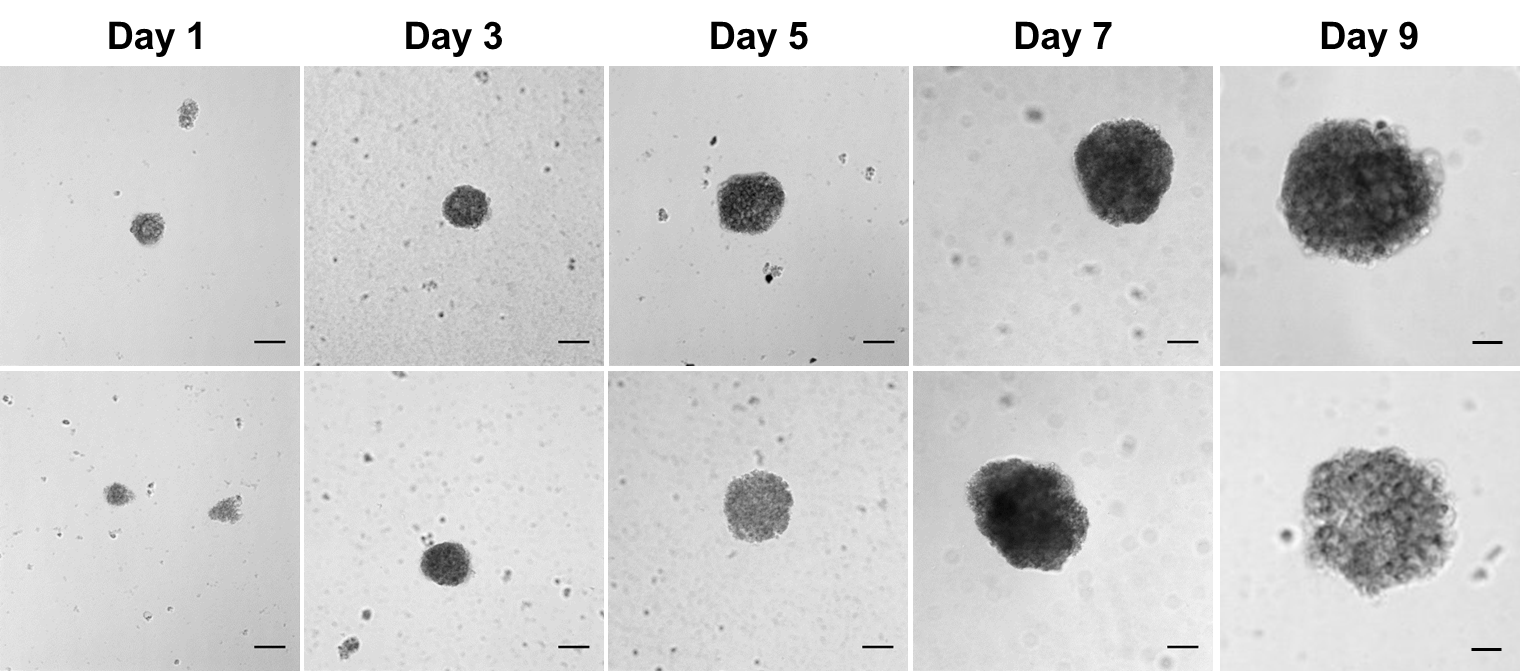


Supplementary Fig 26: Study on tumour mimicking 3D spheroid cultures (SkBr3 cell line).

Details of software’s:

1. Avogadro version number: 1.2.0; Avogadro CMake version file - http://avogadro.cc/
2. Discovery Studio 2020 Client (DS v20.1.0.19295 version); Licensed to Indian Institutes of Technology (IIT), Jodhpur, India
3. OriginPro 8.5; https://www.originlab.com/
4. ImageJ bundled with 64-bit Java 1.8.0_172; https://imagej.nih.gov/ij/
5. BD FACSDiva Software; https://www.bdbiosciences.com/en in/products

/software/instrument-software/bd-facsdiva-software
